# Worldwide Impact of Human Development and Inequality on the Prevalence of Asthma, Rhinoconjunctivitis and Eczema. Global Asthma Network's Ecological Study

**DOI:** 10.1111/cea.70355

**Published:** 2026-06-04

**Authors:** Luis García‐Marcos, Chen‐Yuan Chiang, M. Innes Asher, Guy B. Marks, Asma El Sony, Refiloe Masekela, Karen Bissell, Eamon Ellwood, Philippa Ellwood, Neil Pearce, David P. Strachan, A. Elena Martínez‐Torres, Manuel Sánchez‐Solís, Kevin Mortimer, Eva Morales, The Global Asthma Network Phase I Study Group

**Affiliations:** ^1^ Paediatric Allergy and Pulmonology Units Virgen De la Arrixaca University Children's Hospital, University of Murcia, and IMIB Bio‐Medical Research Institute Murcia Spain; ^2^ International Union Against Tuberculosis and Lung Disease Paris France; ^3^ Division of Pulmonary Medicine, Department of Internal Medicine Wan Fang Hospital, Taipei Medical University Taipei Taiwan; ^4^ Division of Pulmonary Medicine, Department of Internal Medicine, School of Medicine, College of Medicine Taipei Medical University Taipei Taiwan; ^5^ Department of Paediatrics: Child and Youth Health, Faculty of Medical and Health Sciences University of Auckland Auckland New Zealand; ^6^ Respiratory and Environmental Epidemiology University of New South Wales Sydney New South Wales Australia; ^7^ Epidemiological Laboratory (Epi‐Lab) for Public Health, Research and Development Khartoum Sudan; ^8^ Inkosi Albert Luthuli Central Hospital and Department of Paediatrics and Child Health, College of Health Sciences, School of Clinical Medicine University of KwaZulu‐Natal Durban South Africa; ^9^ School of Population Health Faculty of Medical and Health Sciences, University of Auckland Auckland New Zealand; ^10^ Department of Medical Statistics London School of Hygiene & Tropical Medicine London UK; ^11^ Population Health Research Institute St George's, University of London London UK; ^12^ Cambridge Africa, Department of Pathology University of Cambridge Cambridge UK; ^13^ Department of Paediatrics and Child Health School of Clinical Medicine, College of Health Sciences, University of KwaZulu Natal Durban South Africa; ^14^ Department of Respiratory Medicine Aintree University Hospital Liverpool UK; ^15^ Department of Public Health Sciences University of Murcia and IMIB Bio‐Medical Research Institute Murcia Spain; ^16^ Instituto de Salud Carlos III, Spanish Ministry of Health Consortium for Biomedical Research in Epidemiology and Public Health (CIBERESP) Madrid Spain

**Keywords:** asthma, eczema, human development, inequality, Rhinoconjunctivitis

## Abstract

**Background:**

Lower‐income countries have lower asthma prevalence. However, how differences in human development and inequality can explain changes in the prevalence of asthma and allergic diseases is not known.

**Methods:**

The Global Asthma Network Phase I study reported prevalence of asthma and allergic diseases in children (6–7 years), adolescents (13–14 years), and their parents/guardians in 16, 25 and 17 countries. Gini inequality index (GinI) and human development index (HDI), together with mean annual relative humidity and temperature, and latitude, were used as potential explanatory factors of prevalence differences using meta‐regression models, fitted values and heatmaps of prevalence.

**Results:**

GinI and HDI explained some proportion of asthma prevalence variability, which was highest in children (up to ~70% for disease indicators such as current wheeze or symptoms of severe asthma) and lowest in adolescents (~22% for symptoms of severe asthma or asthma ever). Rhinoconjunctivitis prevalence variability was poorly explained by covariates (from ~53% for current rhinoconjunctivitis among children—an exception—to none). Eczema indicators were explained in a range from ~60% in children (current eczema symptoms and symptoms of severe eczema) to ~12% in adolescents (current eczema symptoms). Overall, heatmaps showed areas of higher prevalence in the intersection of high GinI and high HDI values.

**Conclusions:**

HDI and Gini explain part of the worldwide variability in the prevalence of asthma, rhinoconjunctivitis, and eczema. This explanatory power is highest for asthma and lowest for rhinoconjunctivitis. Lower‐resourced communities in highly developed countries show the greatest hazard, particularly for asthma.

## Key Message Box

- The Global Asthma Network (GAN) surveyed children, adolescents, and adults in many centres worldwide.
- Ecological factors may explain differences in the prevalence of asthma, rhinoconjunctivitis, and eczema between centres.
- Human development and inequality partly explain between‐centre variability, especially for asthma prevalence.

## Graphical abstract

Global Asthma Network Phase I surveyed children, adolescents, and adults worldwide to assess the prevalence of asthma, rhinoconjunctivitis, and eczema. Human Development Index (HDI) and Gini inequality index (GinI) were important explanatory factors for prevalence variability of these conditions, with hotspots in areas with high HDI and high GinI.

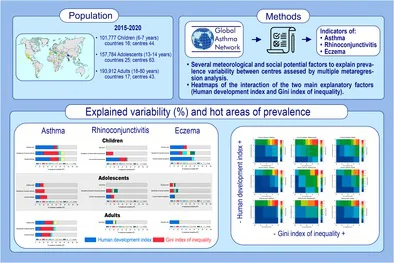

## 1 Introduction

Asthma and allergies are highly prevalent conditions worldwide. However, their prevalence varies considerably [1, 2] and the reasons for this variability are poorly understood. Although known risk factors acting on individuals may explain some of those differences, an ecological perspective could add important insight into the issue. In Phase III of the International Study of Asthma and Allergies in Childhood (ISAAC), carried out between 2001 and 2003 [3], several ecological analyses of various factors were performed, including Gross National Income (GNI), diet, immunisation, climate (outdoor and indoor), tobacco smoking, air pollution (PM10), paracetamol and antibiotic use, and pollen. Some significant associations were found at the centre level for factors such as GNI and per capita paracetamol sales (positive) or PM10 (negative) [4].

In the Global Asthma Network (GAN) Phase I study, performed between 2015 and 2020, the highest GNI category was generally associated with higher prevalence of asthma, rhinoconjunctivitis and eczema in children, adolescents and adults [1, 2]. However, GNI is a purely national income measure and as such has limitations [5]. It does not, for example, capture economic activity which comes from informal or subsistence activities or reflect inequalities in the income distribution within countries, nor include important aspects of the socioeconomic situation within geographical areas, such as inequality or education, which might be important for a better understanding of the epidemiology of asthma and allergies worldwide. Human Development Index (HDI) is a summary measure of average achievement in key dimensions of human development, which include life expectancy, schooling and standard of living [6]. Previously, an analysis of the association of the HDI and the Gini index of inequality (GinI) with asthma prevalence was performed in Latin America on partial data from ISAAC Phase III [7, 8]. In both children and adolescents, HDI was negatively associated with asthma prevalence, while GinI was positively associated. However, in a separate analysis of only Brazilian surveys, neither HDI nor GinI showed such an association [9]. More recently, a GAN Phase I study in six Kosovo cities found a negative association between asthma prevalence and HDI [10]. Those discrepancies probably highlight the need for a high sample size, together with enough variability of social indices. GAN Phase I, being a global study, may offer an adequate sample size and variability. Furthermore, studies performed so far did not show to what extent HDI or GinI could explain the variability of the prevalence of asthma between centres. Additionally, and to the best of our knowledge, no information is available on how those two social indices could be related to rhinoconjunctivitis or eczema, or to what extent they might explain their prevalence variability.

The aim of the present paper is to show how much of the worldwide variability of several indicators of asthma, rhinoconjunctivitis and eczema can be explained by HDI and GinI in the global GAN Phase I study.

## 2 Methods

The GAN Phase I study objectives and methods have been described previously [11]. Briefly, GAN Phase I is a centre‐based cross‐sectional international study based on responses to written questionnaires delivered in schools to children 6–7 years of age and their parents (both optional), and adolescents 13–14 years old (compulsory).

### 2.1 Questionnaires

The definitions of indicators of asthma, rhinoconjunctivitis and eczema were based on the written questionnaires, which were completed at home by the parents of children (children and parents' questionnaires) and by adolescents at school. The original questionnaire was in English. Some centres included the optional video‐questionnaire on asthma in adolescents [12]. Previously validated ISAAC Phase I core questionnaires on asthma, rhinoconjunctivitis and eczema symptoms had been translated into the local languages in accordance with the ISAAC protocol [13]. Core questionnaires were identical in ISAAC Phases I and III and in GAN Phase I.

### 2.2 Definitions

All definitions follow those already used in GAN Phase I and already published [2], as follows:

#### 2.2.1 Indicators of Asthma

“Current wheeze” was defined by an affirmative answer to the question “Have you (has your child) had wheezing or whistling in the chest in the past 12 months?” “Symptoms of severe asthma” were defined when current asthma symptoms included ≥ 4 attacks of wheeze, or > 1 night per week sleep disturbance from wheeze, or wheeze affecting speech in the same period. “Asthma ever” was defined as a positive answer to the question “Have you (Has your child) ever had asthma?”

#### 2.2.2 Indicators of Rhinoconjunctivitis

“Current rhinoconjunctivitis symptoms” was defined from the affirmative answers to two different questions in the written questionnaire: “In the past 12 months, have you (has this child) had a problem with sneezing, or a runny or blocked nose when he/she did not have a cold or the flu?” and “In the past 12 months, has this (child's) nose problem been accompanied with itchy‐watery eyes” “Symptoms of severe rhinoconjunctivitis” was defined by the response “a lot” to the question “In the past 12 months, how much did this (child's) nose problem interfere with your (his/her) daily activities? (Not at all, a little, a moderate amount, a lot)”. “Hay fever ever” was defined as a positive answer to: “Have you (Has your child) ever had hay fever?”

#### 2.2.3 Indicators of Eczema

“Current eczema symptoms” was defined as a positive answer to the two questions: “Have you (has this child) had this itchy rash [defined in a previous question] at any time in the past 12 months?” and “Has this itchy rash at any time affected any of the following places: The folds of the elbows, behind the knees, in front of the ankles, under the buttocks, or around the neck, ears or eyes?” “Symptoms of severe eczema” was defined as a positive answer to awakening one or more times per week in the question “In the past 12 months, how often, on average, have you (has this child) been kept awake at night by this itchy rash? (Never, less than one night per week, one or more nights per week)”. “Eczema ever” was defined as an affirmative answer to: “Have you (Has your child) ever had eczema?”

#### 2.2.4 Social Indexes

HDI was obtained from the Global Data Lab website repertoire for the specific region of each GAN centre [14] and checked against the country values reported by the World Bank [15]. The GinI was obtained from the World Bank website [16]. GinI measures the deviation of the income or consumption of a given economy from an equal distribution. When GinI is 0, the specific economy is in perfect equality; when it is 100, the inequality is absolute [17]. As reliable information on GinI was only available at the country level, and as the number of countries with multiple centres was very small, the country index was imputed to all GAN centre(s) within each country. Both for HDI and GinI, the value for 2020 (or closest, when no data were available for that year) was used for consistency with prior analyses of GAN data related to GNI [1, 2].

#### 2.2.5 Meteorological and Geographical Variables

To control for meteorological and geographical factors that might behave as non‐modifiable risk factors which might be associated both with social indexes and disease indicators, latitude, annual relative humidity and mean annual temperature were included in the analyses. Humidity and temperature were taken from the Climate Data website [18] as shown at the moment of the accession date (2022) for the geographical areas of the GAN centres. Latitude was computed as the absolute value of the distance of each centre from the equator in decimal degrees with a precision of two decimal places (city level).

### 2.3 Sample Size and Power

The study data collection began in January 2015 and finished in May 2020. All students of the target age within schools (selected randomly when their number exceeded that required to recruit the planned sample) were invited to participate and were selected by grade or by age. A sample size of 3000 was sought in each age group (with a minimum of 1000 deemed acceptable) to have enough power (> 90%) to detect (at a significance level of 0.01) prevalence differences of 5% at the expected asthma prevalence, and to allow for testing multiple hypotheses. Additional details of the sample size and power are described elsewhere [11, 19, 20]. High participation rates were sought (response rate of at least 80% for adolescents and 70% for children) and achieved [3]. Centres with a participation rate lower than 50% were excluded.

### 2.4 Data Handling and Analysis

Data handling from each centre has been described in detail elsewhere [11]. A uniform approach to data processing, checking, and analysis was used by means of Stata Statistical Software versions 13 and 15 (College Station, TX, USA). Participation and prevalence calculations have also been previously reported [11].

After logit conversion of the proportion (those with positive answers to the question defining an indicator over all respondents of at least one of the questions) and standard error of each disease indicator in each centre and age group, a random effects meta‐analysis (restricted maximum likelihood method) was performed to check for significant heterogeneity as measured by *T* ^2^, *Q* and *I* ^2^.

Meta‐analyses of every indicator within each age group were followed by simple random‐effects meta‐regressions (residual maximum likelihood method) including the following covariates: HDI (range 0 to 1) [6], mean annual relative humidity (%), mean annual temperature (°C), and latitude (absolute distance from Equator) at the centre level; and GinI (range 0–100) [17] at the country level. Because the number of countries with three or more centres was small (3/16, 4/25 and 3/17 for children, adolescents, and adults, respectively), a multilevel structure of the data was not assumed. The adjusted *R* ^2^ of the meta‐regression was interpreted as a surrogate of the proportion of between‐centre prevalence variability of each disease indicator that could be explained by the main effects of each covariate (simple model) or by all covariates combined (fully adjusted model). Limits for 95% confidence intervals were obtained via nonparametric bootstrap resampling (1,000 replications) to account for the uncertainty and nonlinearity of the measure. Due to the relatively low number of centres, we did not assess covariate interactions. In particular, we did not include an interaction term between HDI and GinI as we wanted to show that interaction (if any) in the heatmaps described below. To refine the potential explanatory effect of covariates, a third meta‐regression (best adjusted model) was performed, including only those covariates which showed some association with the disease indicator (*p* < 0.2) in the fully adjusted model. When this model was statistically significant (*p* < 0.05), each of the covariates was included step by step to put together the best adjusted model, provided they did not reduce at all the *R* ^2^ of the model. Adjusted associations were indicated by the corresponding exponent (aExp). Using the regression equations of the best adjusted models (when both HDI and GinI were included), fitted values (predicted prevalence) were calculated, and residual and regression plots were drawn. Additionally, heatmaps of predicted prevalence, HDI and GinI were generated for the observed ranges in the study. Potential correlation between HDI and GinI was explored by the Spearman test.

### 2.5 Ethics

All centres obtained approval from their corresponding ethics committee. They determined the method of obtaining consent (either passive or written) from parents or caregivers, though GAN recommended passive consent [21]. Adolescents assented to participating in the survey.

### 2.6 Role of Funding Source

None.

## 3 Results

Prevalence information on asthma, rhinoconjunctivitis and eczema indicators was obtained from 44 (children), 63 (adolescents) and 43 (adults) centres from 16, 25 and 17 countries, respectively. The prevalence range of all disease indicators was wide and showed significant true heterogeneity in the meta‐analyses. Potential explanatory covariates of this variability included in meta‐regression analyses (HDI, GinI, mean annual relative humidity, mean annual temperature and latitude) varied widely (Tables 1, 2, 3).

**TABLE 1:** Summary of variables included in meta‐regression analyses in children: NUmber of countries and centres; ranges of potential explanatory covariates of prevalence variability between centres (HDI, GINI, mean annual relative humidity, temperature and latitude); summary and range (in parentheses) of the prevalence of each disease indicator; and the corresponding heterogeneity tests from the random effects metanalyses (RML method).

Countries	Centres	HDI^a^	GINI^b^	Mean annual relative humidity (%)	Mean annual temperature (°C)	Latitude (decimal degrees)	Disease indicator	Summary prevalence (%) (range)	T^2^	Q	*p*	I^2^ (%)
16	44	0.594–0.945	29.9–48.2	22.8–84.1	2.2–27.7	7.27–57.15	Current wheeze	7.9 (3.5–23.2)	0.5956	1939.18	< 0.001	99.1
							Symptoms of severe asthma	3.3 (0.4–13.2)	0.5880	1162.82	< 0.001	98.0
							Asthma ever	5.4 (3.1–29.3)	1.027	3860.78	< 0.001	99.4
							Current rhinoconjunctivitis	6.0 (1.5–24.0)	0.7936	3046.54	< 0.001	99.2
							Symptoms of severe rhinoconjunctivitis	0.5 (0.0–2.5)	0.4065	288.08	< 0.001	84.5
							Hay fever ever	9.9 (0.0–51.2)	0.6643	6713.79	< 0.001	99.3
							Current eczema	4.9 (0.4–15.7)	0.4434	1852.68	< 0.001	98.0
							Symptoms of severe eczema	0.6 (0.0–2.1)	0.3403	246.98	< 0.001	84.3
							Eczema ever	8.1 (1.1–47.5)	1.1985	13110.01	< 0.001	99.6

^a^ Human development index (range 0–1).

^b^ Gini index of inequality (range 0–100).

**TABLE 2:** Summary of variables included in meta‐regression analyses in adolescents: NUmber of countries and centres; ranges of potential explanatory covariates of prevalence variability between centres (HDI, GINI, mean annual relative humidity, temperature and latitude); summary and range (in parentheses) of the prevalence of each disease indicator; and the corresponding heterogeneity tests from the random effects metanalyses (RML method).

Countries	Centres	HDI^a^	GINI^b^	Mean annual relative humidity (%)	Mean annual temperature (°C)	Latitude (decimal degrees)	Disease indicator	Summary prevalence (%) (range)	T^2^	Q	*p*	I^2^ (%)
25	63	0.457– 0.945	28.5– 63	22.8– 84.4	2.2– 29.9	0.23– 57.153	Current wheeze	9.9 (0.9–21.4)	0.4438	3267.11	< 0.001	99.1
							Symptoms of severe asthma	4.5 (0.5–14.7)	0.5014	2318.13	< 0.001	98.4
							Asthma ever	8.3 (0.3–3.0)	0.7522	5639.63	< 0.001	99.4
							Current rhinoconjunctivitis	12.8 (4.8–30.1)	0.2108	3227.49	< 0.001	98.4
							Symptoms of severe rhinoconjunctivitis	0.7 (0.1–10.0)	0.4454	668.37	< 0.001	90.3
							Hay fever ever	13.7 (3.7–44.0)	0.7006	10974.01	< 0.001	99.5
							Current eczema	5.5 (1.4–18.5)	0.3300	3126.75	< 0.001	98.0
							Symptoms of severe eczema	0.8 (0.0–4.7)	0.3850	769.46	< 0.001	90.5
							Eczema ever	7.5 (1.7–38.4)	0.8203	9911.64	< 0.001	99.4

^a^ Human development index (range 0–1).

^b^ Gini index of inequality (range 0–100).

**TABLE 3:** Summary of variables included in meta‐regression analyses in **adults**: NUmber of countries and centres; ranges of potential explanatory covariates of prevalence variability between centres (HDI, GINI, mean annual relative humidity, temperature and latitude); summary and range (in parentheses) of the prevalence of each disease indicator; and the corresponding heterogeneity tests from the random effects metanalyses (RML method).

Countries	Centres	HDI^a^	GINI^b^	Mean annual relative humidity (%)	Mean annual temperature (°C)	Latitude (decimal degrees)	Disease indicator	Summary prevalence (%) (range)	T^2^	Q	*p*	I^2^ (%)
17	43	0.594– 0.945	28.5– 53.5	22.8– 84.4	2.2– 27.7	7.38– 57.15	Current wheeze	7.0 (0.9–32.7)	0.5100	4826.83	< 0.001	99.3
							Symptoms of severe asthma	2.7 (0.2–20.9)	0.6971	2177.39	< 0.001	98.7
							Asthma ever	3.6 (0.5–29.0)	0.9370	7306.15	< 0.001	99.4
							Current rhinoconjunctivitis	*N*.A				
							Symptoms of severe rhinoconjunctivitis	*N*.A				
							Hay fever ever	11.7 (2.8–45.7)	0.9310	19252.59	< 0.001	99.8
							Current eczema	*N*.A				
							Symptoms of severe eczema	*N*.A				
							Eczema ever	7.0 (1.6–29.5)	0.8060	8741.07	< 0.001	99.7

Abbreviation: N.A., Not available.

^a^ Human development index (range 0–1).

^b^ Gini index of inequality (range 0–100).

### 3.1 Explanation of the Variability of Asthma Prevalence

**FIGURE 1:**
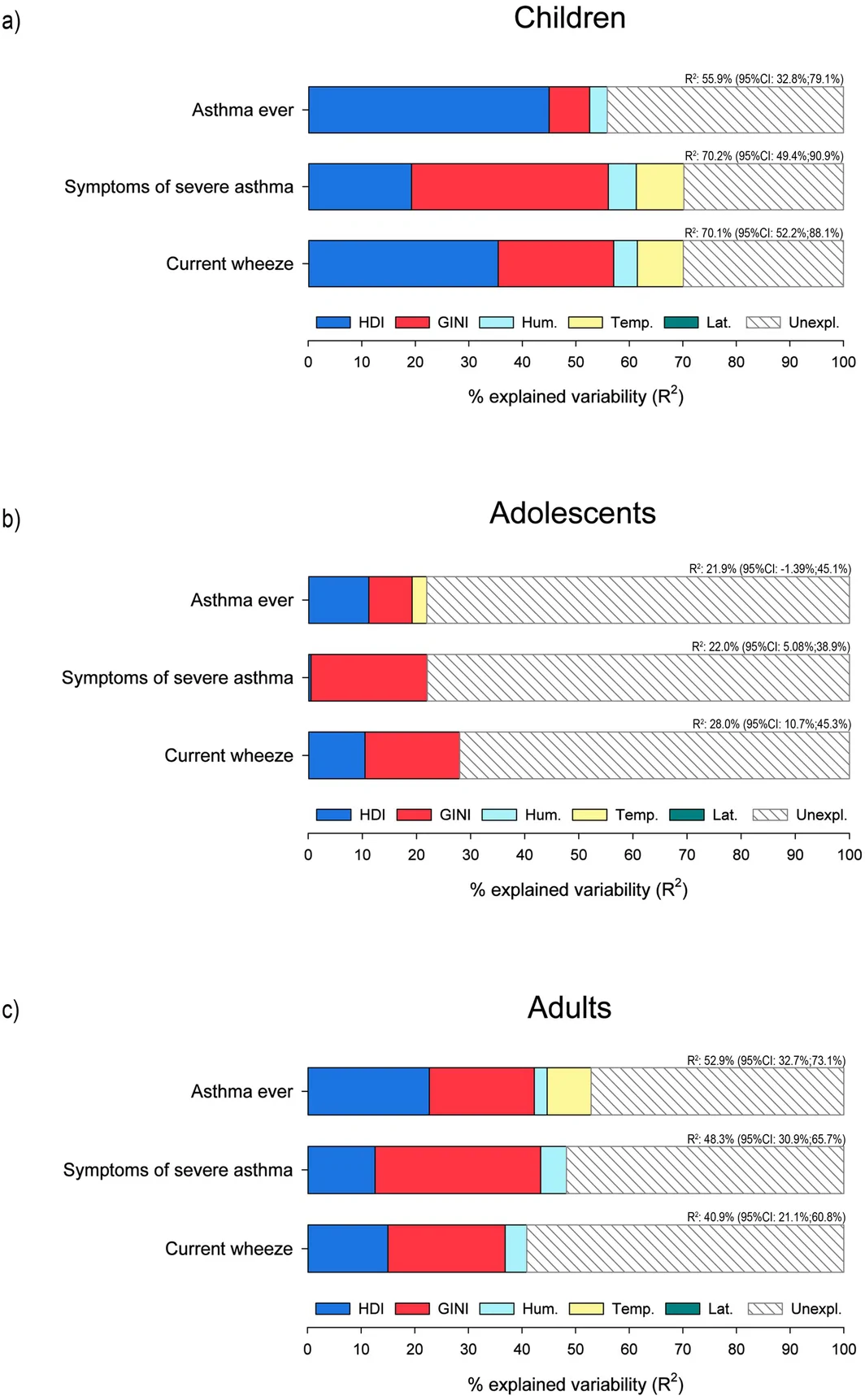
Best adjusted models of meta‐regression analyses of the different asthma indicators among (a) children, (b) adolescents and (c) adults: Potential of covariates to explain the variability (*R* ^2^ [95% CI]) of prevalence between centres. HDI: Human development index; GinI: GinI index of inequality; Hum.: Mean relative humidity (%); Temp.: Mean annual temperature (°C); Lat. (decimal degrees); Unexpl.: Variability not explained by covariates in the best adjusted meta‐regression analysis.

Covariates explained a considerable proportion of the variability of the prevalence of asthma indicators, which was highest in children and lowest in adolescents: Ranging from ~70% for current wheeze and symptoms of severe asthma in children to ~22% for symptoms of severe asthma and asthma ever among adolescents in the lower range (Tables S1–S3; Figure 1). Overall, covariates explained more variability among children than among adolescents or adults, although among the latter, it was relatively high (ranging from ~41% to ~53%) for all indicators (Tables S1–S3; Figure 1). Overall, HDI and GinI explained more variability than humidity or temperature. Latitude did not show any explanatory effect. Associations of covariates with the prevalence of asthma indicators were positive. Thus, higher levels of human development (higher HDI) and increased inequality (higher GinI) were independently associated with higher prevalence of asthma indicators (Tables S1–S3). There were no significant correlations between HDI and GinI in any age group (children: rho = −0.20, *p* = 0.188; adolescents: rho = −0.06, *p* = 0.617; and adults: rho = 0.07, *p* = 0.654).

### 3.2 Explanation of the Variability of Rhinoconjunctivitis Prevalence

**FIGURE 2:**
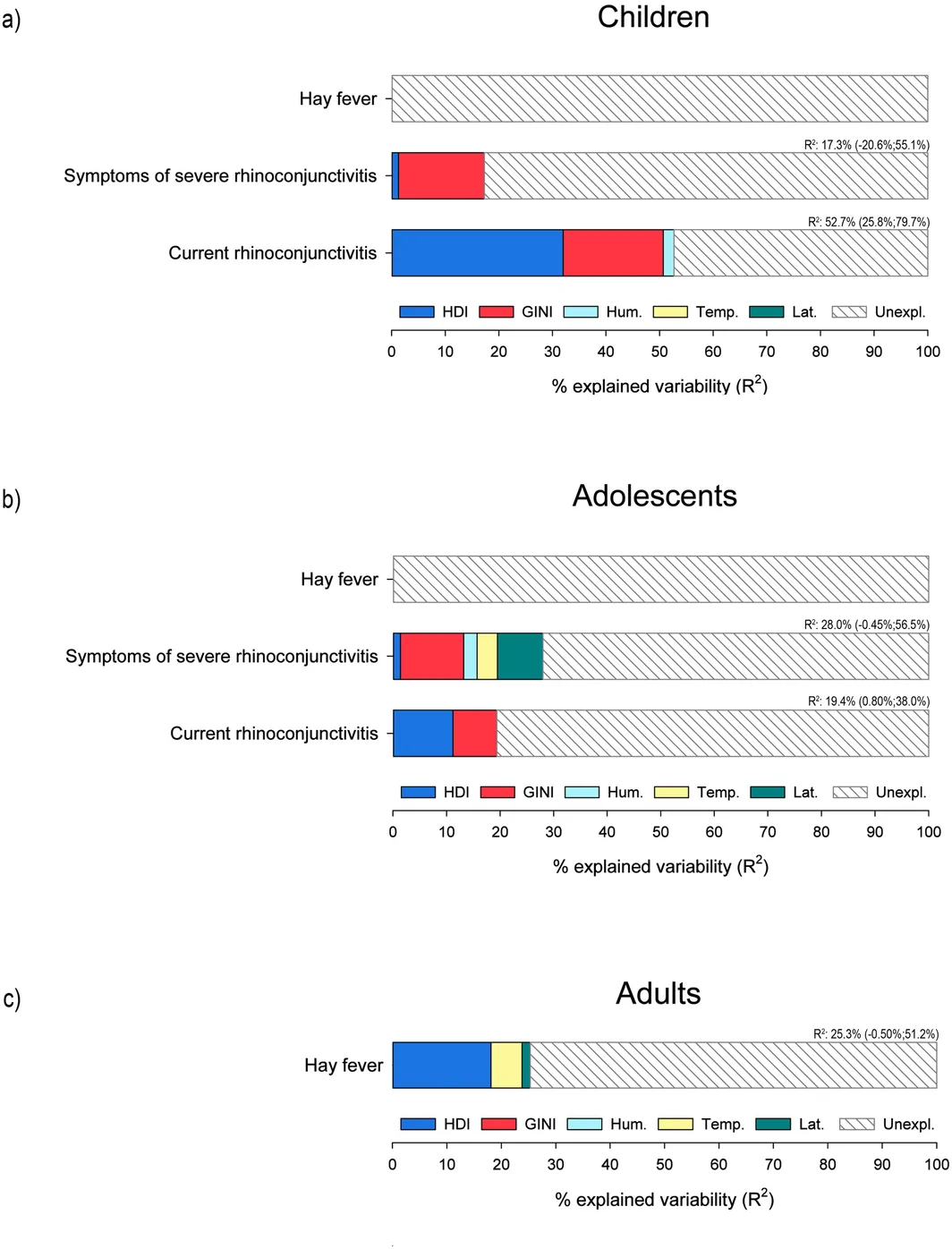
Best adjusted models of meta‐regression analyses of the different rhinoconjunctivitis indicators among (a) children, (b) adolescents and (c) adults: Potential of covariates to explain the variability (*R* ^2^ [95% CI]) of prevalence between centres. HDI: Human development index; GinI: GinI index of inequality; Hum.: Mean relative humidity (%); Temp.: Mean annual temperature (°C); Lat. (decimal degrees); Unexpl.: Variability not explained by covariates in the best adjusted meta‐regression analysis.

Covariates did not well explain the variability in prevalence of rhinoconjunctivitis indicators between centres. The combination of covariates explained from ~53% variability (for current rhinoconjunctivitis among children, which was an exception; Figure 2) to none (Tables S4–S6). For adults, only “hay fever” was available, and covariates showed some potential to explain its variability (~25%) (Table S6; Figure 2c).

### 3.3 Explanation of the Variability of Eczema Prevalence

**FIGURE 3:**
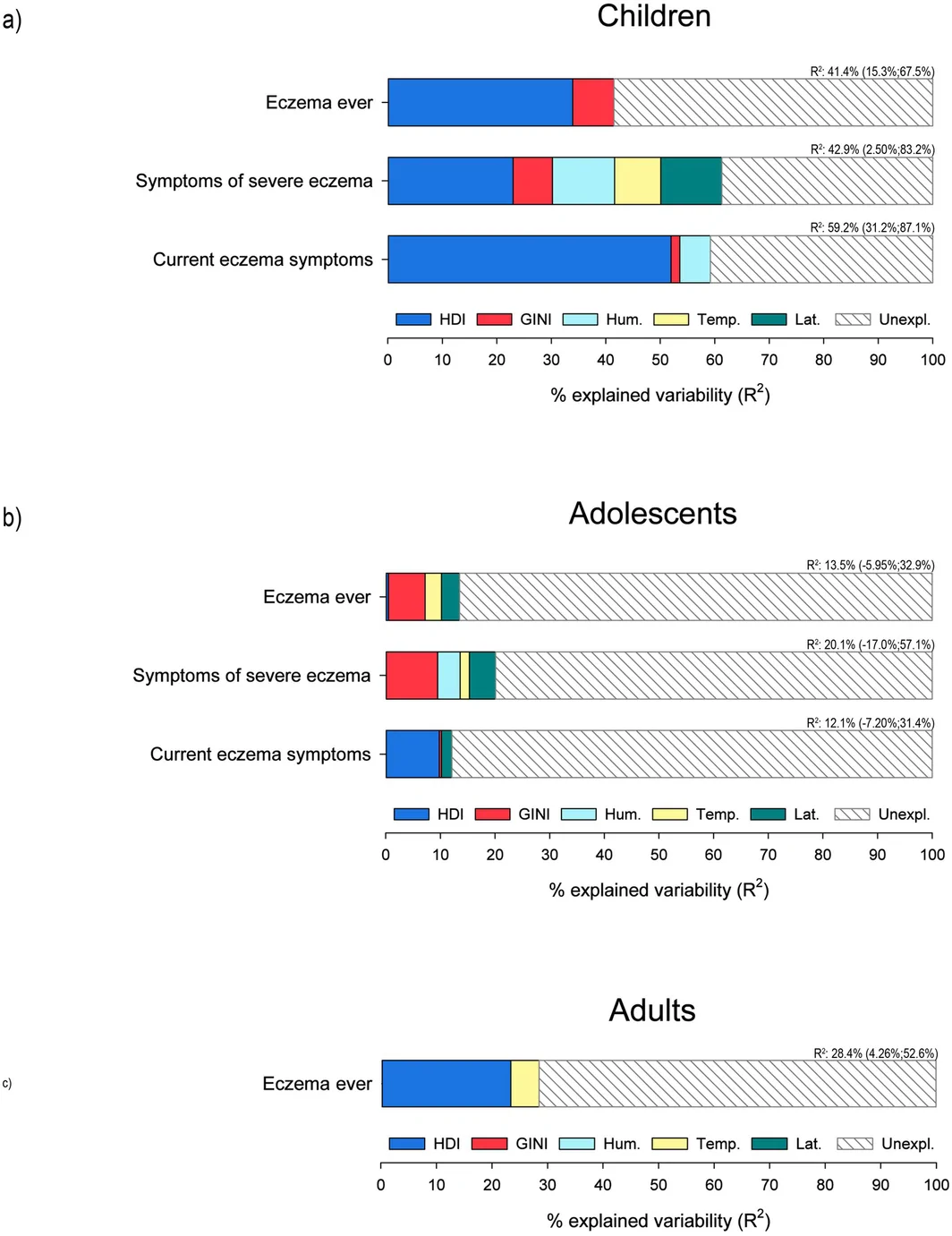
Best adjusted models of meta‐regression analyses of the different eczema indicators among (a) children, (b) adolescents and (c) adults: Potential of covariates to explain the variability (*R* ^2^ [95% CI]) of prevalence between centres. HDI: Human development index; GinI: GinI index of inequality; Hum.: Mean relative humidity (%); Temp.: Mean annual temperature (°C); Lat. (decimal degrees); Unexpl.: Variability not explained by covariates in the best adjusted meta‐regression analysis.

The results on eczema indicators among children show intermediate results between those of asthma and rhinoconjunctivitis (Tables S7–S9; Figure 3). The variability of current eczema symptoms in the group of children was explained by the combined covariates HDI, GinI and humidity up to ~60% (Table S7; Figure 3a). This explanatory ability was lower for symptoms of severe eczema (~43%) (Table S8; Figure 3) and eczema ever (~41%) (Table S9; Figure 3a). Variability of eczema indicators in the group of adolescents was not so well explained by the studied covariates, which could only justify ~21% to ~12% of the variability (Tables S7–S9; Figure 3b). For the adult group, only one indicator was measured (eczema ever), and its variability was explained by ~28% by covariates (mainly HDI) (Table S9; Figure 3c). Associations between HDI and GinI with the prevalence of eczema indicators tended to be positive (when present) (Table S7,S8) except for eczema ever, in which HDI was positive and GinI negative (Table S9).

### 3.4 HDI And GinI Zones of Higher Prevalence

We were able to calculate fitted values, draw residual (Figures S1–S3) and regression (Figures S4–S6) plots, and build heatmaps (Figures S7–S9) for most disease indicators except for hay fever (all age groups), symptoms of severe eczema (children and adolescents, not explored in adults), and eczema ever (adults). According to those plots, meta‐regressions performed reasonably well to fit values which could be used to approximate the levels of HDI and GinI of higher prevalence of indicators. Within the range of those two social indices in the study, it was consistently found, in all ages and disease indicators, that the higher fitted prevalences (red areas) were situated in the intersection of HDI range 0.8–0.9 and GinI range 45–50 (Figures S7–S9). The only exception was “eczema ever”, which showed higher prevalence in the intersection area of high HDI and low GinI (Figure S9).

## 4 Discussion

This GAN Phase I study, carried out in three age groups from different centres and countries worldwide, found that substantial variability of the prevalence of indicators of asthma, rhinoconjunctivitis, and eczema might be explained by socioeconomic determinants of health such as HDI and GinI. Also, those two factors tended to explain variability best among children, moderately among adults, and worst among adolescents. As an ecological study, however, those findings should be interpreted with caution and not extrapolated to the individual level.

Overall, the associations of HDI and GinI tended to be positive, indicating a higher prevalence of asthma and allergic diseases in areas of higher development and increased inequality. The associations were strongest for asthma indicators, particularly among children.

Few previous studies have investigated the association between HDI, GinI and asthma and allergic diseases. The study by Fattore et al. [8] which analysed the 6–7‐year‐olds asthma prevalence rates of 31 Latin American centres of ISAAC Phase III, situated those two indices at the first level of a conceptual framework to explore the influence of social determinants of asthma prevalence. Using multiple regression analyses, they found that GinI (positively) and HDI (negatively) were both associated with asthma prevalence at the centre level in the univariate analyses; however, HDI lost significance in the multivariate analyses. GinI could explain up to 48% of the variability of asthma prevalence. Fattore et al. [7] also performed a similar analysis of 48 Latin American centres of the same ISAAC phase in adolescents, finding similar results, although GNI remained marginally significant. GinI and HDI could explain about 35% of centre variability. In the present study, both GinI and HDI showed a positive and independent correlation with asthma prevalence in the three age groups. The discrepancy with Fattore et al. [8] regarding HDI may stem from the wider ranges of this index (and also of GinI) in our study. Additionally, their study is located only in Latin America, while the present one includes centres from multiple locations around the world, probably changing other ecological variables which may interact in different ways. On the other hand, they found that HDI lost its statistical significance in the multivariate analysis. That HDI has a direct correlation with the prevalence of asthma and other allergic diseases is probably in keeping with studies showing a positive association between higher GNI and increased prevalence of asthma and allergies, as was the case in previous reports of GAN Phase I [1, 2]. That higher GINI is correlated with higher asthma prevalence probably reflects a greater disease burden in areas of inequality in countries with high HDI [22, 23]. Interestingly, GinI explains more variability than HDI (Figure 1), perhaps suggesting that social development is responsible for the increase in asthma prevalence whilst poverty increases severity [24]. It is quite possible that, in pockets of poverty within otherwise socially developed areas, factors such as air pollution (outdoor or indoor) from local sources, for example wood stoves [25]; overexposure to certain allergens (such as cockroaches, rats or mice) [26]; and a Western lifestyle (including access to fast food, overweight, etc.) [27]; combined with lower exposure to potential protective factors such as farm‐related material [28], might explain at least in part the present results.

The correlation of asthma prevalence with HDI and GinI in our study is independent of meteorological variables or latitude (which may also be a marker of climate). Although colder winters could increase asthma prevalence in already cold regions [29], ISAAC Phase III did not find any relationship between mean annual temperature, humidity, or latitude (within Europe) and the prevalence of current wheeze at the centre level [4, 30]. The contribution of weather variables or latitude to explain the variability of the prevalence of asthma indicators in the present study was also minimal, as compared to HDI or GinI.

Although we are not aware of any studies reporting associations of HDI or GinI with allergic rhinoconjunctivitis or eczema, it is well established that both conditions are more frequent in high‐income countries [1, 2, 4], and within those countries, it is higher among individuals with higher socioeconomic status (SES) [31, 32]. On the other hand, there is not much information on whether lower SES is associated with more severe rhinoconjunctivitis or eczema beyond the fact that access to medicines or specific immunotherapy could be restricted in low SES populations even inside high‐income countries [33]. However, country GNI or population SES might not be capturing factors included in HDI or GinI, thus comparisons of the present findings with the aforementioned studies should be taken carefully. For instance, in 2020, USA vs. Germany had somewhat higher GNI per capita ($64.700 vs. $59.560; 108%), slightly lower HDI (0.923 vs. 0.948; 97%), but quite different GinI (39.7 vs. 32.4; 123%) [34].

Different from this general trend, the prevalence of “eczema ever” is highest in areas with high HDI but with low GinI. This may reflect some diagnostic bias, as this indicator is related to doctor‐diagnosed eczema. This diagnostic bias might also explain the inability to find good meta‐regression models (except partially for adults) for “hay fever”.

### 4.1 Limitations and Strengths

Given the ecological nature of the study, the exact effect of covariates on individuals cannot be known. Also, HDI and GinI reflect various other factors such as diagnostic awareness (access to health facilities, etc.), protective elements (related to the hygiene hypothesis, etc.), or risk ones (e.g., migration, etc.). In addition, we could not obtain GinI at the centre level and had to impute it at the national level. However, due to the small number of countries with three or more centres, a multilevel analysis was not an option. This may have masked the effect of GinI as an explanatory covariate of the variability of the prevalence of the different disease indicators between centres. However, GinI of the country did show some potential explanatory effect for several of those indicators, especially for asthma. Still, our approach might overestimate the explanatory power and precision of inequality measures because of unmodelled clustering and repeated country‐level covariates. Furthermore, we did not include other ecological variables such as smoking or diet, which could interact with the socioeconomic indexes. However, given the number of centres, we did not think it appropriate to introduce more covariates in the meta‐regression models. The main strength of our study is having data from a unique study using GAN Phase I methodology, including centres worldwide with considerably different prevalence of indicators of asthma, rhinoconjunctivitis and eczema alongside levels of HDI and GinI, allowing for the investigation of their explanatory effects at the ecological level in three different age groups.

## 5 Conclusions

In summary, we found that HDI and GinI can explain some of the variability in the prevalence of several indicators of asthma, rhinoconjunctivitis and eczema worldwide, which is higher for asthma and lowest for rhinoconjunctivitis. Our results also indicate that, at least in asthma (either current wheeze, or symptoms of severe asthma or asthma diagnosis), lower‐resourced communities in highly developed countries are the most at risk.

## Author Contributions

The following individual contributions were made: Conceptualisation M.I.A., K.B., C.‐Y.C., A.E.S., P.E., L.G.‐M., G.M., N.P., D.P.S.; data curation E.E., P.E., L.G.‐M., E.M.; formal analysis L.G.‐M., E.M., M.S.‐S.; investigation L.G.‐M.; methodology M.I.A., C.‐Y.C., P.E., L.G.‐M., N.P., D.P.S.; project administration M.I.A., E.E., P.E.; resources M.I.A., K.M., R.M.; supervision L.G.‐M., M.I.A., C.‐Y.C., K.B., A.E.S., N.P., D.P.S.; validation P.E.; visualisation E.E., P.E.; writing – original draft L.G.‐M., A.E.M.‐T., M.S.‐S., E.M.; writing – review/editing M.I.A., K.B., C.‐Y.C., A.E.S., E.E., P.E., E.M., K.M., N.P., D.P.S., G.M., R.M. and the Global Asthma Network Phase I Study Group; the latter contributed original data to the analyses. Verification of the underlying data was undertaken by L.G.‐M., E.M., A.E.M.‐T., N.P., and D.P.S.

## Funding

The GAN Global Centre in Auckland was funded by The University of Auckland with additional funding from The International Union Against Tuberculosis and Lung Disease, Boehringer Ingelheim NZ, and an Astra Zeneca Educational Grant. The London Data Centre was supported by a PhD studentship [to CR] from the UK Medical Research Council (MRC) (grant number MR/N013638/1) and funding from the European Research Council under the European Union's Seventh Framework Programme (FP7/2007‐2013, ERC grant agreement number 668954). The Murcia Data Centre was supported by the University of Murcia and by Instituto de Salud Carlos III, fund PI17/0170. We thank the NIHR Global Health Research Unit on Lung Health and TB in Africa at LSTM ‐ “IMPALA” for helping to make this work possible. In relation to IMPALA (project reference 16/136/35) specifically: IMPALA was funded by the National Institute for Health Research (NIHR) using UK aid from the UK Government to support global health research. The views expressed in this publication are those of the author(s) and not necessarily those of the NIHR or the UK Department of Health and Social Care. Individual centres involved in GAN Phase I data collection were funded by the following organisations: Argentina; San Francisco by Dr Héctor Badellino; Brazil; Uruguaiana by Dr Marilyn Urrutia Pereira; Cameroon; Yaounde by Elvis Ndikum (95%) and from family, friend (5%); Costa Rica and Nicaragua; partially funded by an unrestricted grant from Astra Zeneca for logistic purposes; India; Bikaner, Chandigarh, Jaipur, Kolkata, Kottayam, Lucknow, Mysuru, New Delhi, Pune, GAN Phase I was undertaken by Asthma Bhawan in India which was supported by Cipla Foundation; Iran; Karaj by the Alborz University of Medical Sciences; Kosovo; Gjakova by the Municipality of Gjakova and the Directorate for Health and Education; Mexico; all centres by Astra Zeneca and COMPEDIA (Colegio Mexicano de Pediatras Especialistas en Inmunologia Clinica y Alergia) educational grants; Puerto Vallarta by Centro Universitario de la Costa, Universidad de Guadalajara; New Zealand; Auckland by Asthma Charitable Trust; Nigeria; Ibadan funded by NIHR (IMPALA grant Ref 16/136/35) using UK aid from the UK Government to support GHR; Poland; Katowice by the Medical University of Silesia; South Africa; Cape Town by the South African Medical Research Council and by the Allergy Society of South Africa for Heather Zar; Syria; Damascus by the Syrian Private University and Lattakia by The Medical National Syndicate; Spain; Cartagena and Bilbao funded by Instituto de Salud Carlos III (grants PI17/00179, PI17/00694, PI17/00756), Cantabria by Instituto de Investigación Sanitaria Valdecilla (IDIVAL) de Cantabria PRIMVAL 17/01 y 18/01, Salamanca by Gerencia Regional de Salud de la Junta de Castilla y León (grant GRS 1239b/16) and Sociedad Española de Inmunología Clínica, Alergología y Asma Pediátrica and A Coruña by María José Jove foundation; Sri Lanka; Anuradhapura and Peradeniya by the University of Peradeniya, Sri Lanka.

## Conflicts of Interest

K.M. reports receiving advisory board fees from AstraZeneca, outside the submitted work. G.B.M. reports grants and non‐financial support from AstraZeneca and grants from GlaxoSmithKline Australia and Novartis Australia, outside the submitted work. All other authors declare no competing interests.

## Supporting information


**Data S1:** Table S1. Impact of the different moderators on the variability (adjusted *R* ^2^) of the prevalence of current wheeze between centres. Simple (crude) and multiple meta‐regression analyses (full and best adjusted models).
**Table S2:** Impact of the different moderators on the variability (adjusted R2) of the prevalence of symptoms of severe asthma between centres. Simple (crude) and multiple meta‐regression analyses (full and best adjusted models).
**Table S3:** Impact of the different moderators on the variability (adjusted R2) of the centre prevalence of asthma ever. Simple (crude) and multiple meta‐regression analyses (full and best adjusted models).
**Table S4:** Impact of the different moderators on the variability (adjusted R2) of the prevalence of current rhinoconjunctivitis symptoms between centres. Simple (crude) and multiple meta‐regression analyses (full and best adjusted models).
**Table S5:** Impact of the different moderators on the variability (adjusted R2) of the prevalence of symptoms of severe rhinoconjunctivitis between centres. Simple (crude) and multiple meta‐regression analyses (full and best adjusted models).
**Table S6:** Impact of the different moderators on the variability (adjusted R2) of the centre prevalence of hay fever ever. Simple (crude) and multiple meta‐regression analyses (full and best adjusted models).
**Table S7:** Impact of the different moderators on the variability (adjusted R2) of the prevalence of current eczema symptoms between centres. Simple (crude) and multiple meta‐regression analyses (full and best adjusted models).
**Table S8:** Impact of the different moderators on the variability (adjusted R2) of the prevalence of symptoms of severe eczema between centres. Simple (crude) and multiple meta‐regression analyses (full and best adjusted models).
**Table S9:** Impact of the different moderators on the variability (adjusted R2) of the prevalence of eczema ever between centres. Simple (crude) and multiple meta‐regression analyses (full and best adjusted models).
**Figure S1:** Residual plots of “current wheeze”, “symptoms of severe asthma” and “asthma ever” in children, adolescents and adults after applying the best adjusted models of the meta‐regression analyses depicted in Tables S1–S3.
**Figure S2:** Residual plots of “current rhinoconjunctivitis symptoms” and “symptoms of severe rhinoconjunctivitis” in children and adolescents after applying the best adjusted models of the meta‐regression analyses depicted in Tables S4–S5. (Those two markers were not available in adults, and no regression, which included HDI and GinI, could be fitted for the common one in the three age groups, i.e., “hay fever”).
**Figure S3:** Residual plots of “current eczema symptoms” and “eczema ever” in children and adolescents after applying the best adjusted models of the meta‐regression analyses depicted in Table S7 and Table S9. (No regression, which included HDI and GinI, could be fitted for “symptoms of severe eczema” in those age groups and was not available in adults. GinI was not a significant covariate in the best adjusted model for “eczema ever” in adults).
**Figure S4:** Regression plots of “current wheeze”, “symptoms of severe asthma” and “asthma ever” in children, adolescents and adults after applying the best adjusted models of the meta‐regression analyses depicted in Tables S1–S3.
**Figure S5:** Regression plots of “current rhinoconjunctivitis symptoms” and “symptoms of severe rhinoconjunctivitis” in children and adolescents after applying the best adjusted models of the meta‐regression analyses depicted in table S4,S5. (Those two markers were not available in adults, and no regression, which included HDI and GinI, could be fitted for the common one in the three age groups, i.e., “hay fever”).
**Figure S6:** Regression plots of “current eczema symptoms” and “eczema ever” in children and adolescents after applying the best adjusted models of the meta‐regression analyses depicted in Table S7 and S9. (No regression, which included HDI and GinI, could be fitted for “symptoms of severe eczema” in those age groups and was not available in adults. GinI was not a significant covariate in the best adjusted model for “eczema ever” in adults).
**Figure S7:** Heatmap of areas of high and low prevalence of “current wheeze”, “symptoms of severe asthma” and “asthma ever” in children, adolescents and adults after applying the best adjusted models of the meta‐regression analyses to the range of values of HDI and GinI in the study. The prevalence range of the specific disease marker is one of the fitted values.
**Figure S8:** Heatmap of areas of high and low prevalence of “current rhinoconjunctivitis symptoms” and “symptoms of severe rhinoconjunctivitis” in children and adolescents after applying the best adjusted models of the meta‐regression analyses to the range of values of HDI and GinI in the study. The prevalence range of the specific disease marker is one of the fitted values. (Both “rhinoconjunctivitis” markers were not available in adults, and no regression, which included HDI and GinI, could be fitted for the common one in the three age groups, i.e., “hay fever”).
**Figure S9:** Heatmap of areas of high and low prevalence of “current eczema symptoms” and “eczema ever” in children and adolescents after applying the best adjusted models of the meta‐regression analyses to the range of values of HDI and GinI in the study. The prevalence range of the specific disease marker is one of the fitted values. (No regression, which included HDI and GinI, could be fitted for “symptoms of severe eczema” in those age groups and was not available in adults. GinI was not a significant covariate in the best adjusted model for “eczema ever” in adults).

## Data Availability

The study protocol including a recommended informed consent form and statistical analysis plan are in the public domain. The GAN Phase I data, including de‐identified individual participant data, will be made available on the Global Asthma Network website http://www.globalasthmanetwork.org/ within 12 months of all GAN Phase I analyses being published. Access will require a formal request, a written proposal and a signed data access agreement.

## Appendix A Global Asthma Network Study Group

**Global Asthma Network Steering Group:** MI Asher, Department of Paediatrics: Child and Youth Health, Faculty of Medical and Health Sciences, University of Auckland, Private Bag 92,019, Auckland, New Zealand; K Bissell, School of Population Health, Faculty of Medical and Health Sciences, University of Auckland, Auckland, New Zealand; C‐Y Chiang, International Union Against Tuberculosis and Lung Disease, Paris, France; and Division of Pulmonary Medicine, Department of Internal Medicine, Wan Fang Hospital, Taipei Medical University; and Division of Pulmonary Medicine, Department of Internal Medicine, School of Medicine, College of Medicine, Taipei Medical University111 Hsin‐Long Road, Section 3, Taipei, 116, Taiwan; A El Sony, Epidemiological Laboratory for Public Health and Research, Khartoum 3 Block3‐Building 11, Khartoum, Sudan; P Ellwood, Department of Paediatrics: Child and Youth Health, Faculty of Medical and Health Sciences, Private Bag 92,019, University of Auckland, Auckland, New Zealand; L García‐Marcos, Paediatric Allergy and Pulmonology Units, Virgen de la Arrixaca University Children‘s Hospital, University of Murcia and IMIB Bioresearch Institute, Murcia; and ARADyAL Allergy Network, Edificio Departamental‐Laib, Avenida Buenavista s/n, 30,120 El Palmar, 30,394 Murcia Spain; GB Marks, Respiratory & Environmental Epidemiology, University of New South Wales, Goulburn St, Sydney 2085, Sydney, Australia; K Mortimer, Liverpool School of Tropical Medicine, Pembroke Place, Liverpool L3 5QA, United Kingdom; N Pearce, Department of Medical Statistics, London School of Hygiene & Tropical Medicine, Keppel Street, London WC1E 7HT, United Kingdom; DP Strachan, Population Health Research Institute, St George's, University of London, Cranmer Terrace, London SW17 0RE, United Kingdom.

Global Asthma Network International Data Centres:

**GAN Global Centre:** P Ellwood, E Ellwood, MI Asher, Department of Paediatrics: Child and Youth Health, Faculty of Medical and Health Sciences, Private Bag 92,019, University of Auckland, Auckland, New Zealand.

**Murcia, Spain:** L García‐Marcos, Paediatric Allergy and Pulmonology Units, Virgen de la Arrixaca University Children‘s Hospital, University of Murcia and IMIB Bioresearch Institute, Murcia; and ARADyAL Allergy Network, Edificio Departamental‐Laib, Murcia, Spain V Perez‐Fernández, Department of Paediatrics, University of Murcia; and IMIB Bio‐health Research Institute, Murcia, Edificio Departamental‐Laib, Avenida Buenavista s/n, 30,120 El Palmar, 30,394 Murcia Spain E Morales, Department of Public Health Sciences, University of Murcia, and IMIB Bio‐health Research Institute, Murcia, Edificio Departamental‐Laib, Avenida Buenavista s/n, 30,120 El Palmar, 30,394 Murcia, Spain A Martinez‐Torres, Paediatric Allergy and Pulmonology Units and Nurse Research Group, Virgen de la Arrixaca University Children‘s Hospital, University of Murcia and IMIB Bio‐health Research Institute, Murcia, Edificio Departamental‐Laib, Avenida Buenavista s/n, 30,120 El Palmar, 30,394 Murcia, Spain.

**London, United Kingdom:** DP Strachan, Population Health Research Institute, St George's, University of London, Cranmer Terrace, London SW17 0RE, United Kingdom N Pearce, S Robertson, CE Rutter, Department of Medical Statistics, London School of Hygiene & Tropical Medicine, Keppel Street, London WC1E 7HT, United Kingdom RJ Silverwood, Department of Medical Statistics, London School of Hygiene & Tropical Medicine, Keppel Street, London WC1E 7HT, United Kingdom and Centre for Longitudinal Studies, UCL Social Research Institute, University College London, 20 Bedford Way, London WC1H 0AL, United Kingdom.

**Global Asthma Network Principal Investigators:** Argentina: H Badellino, Clinica Regional Del Este (San Francisco); Brazil: M Urrutia‐Pereira, Federal University of Pampa, UNIPAMPA (Uruguaiana); Cameroon: AE Ndikum, The University of Yaounde 1 (Yaounde); Chile: J Mallol, University of Santiago de Chile (USACH) (South Santiago); Costa Rica: ME. Soto‐Martínez, Hospital Nacional de Niños “Dr. Carlos Saénz Herrera”, Caja Costarricense Seguro Social—Universidad de Costa Rica, San José (Costa Rica); Ecuador: A Cabrera Aguilar, Respiraclinic (Quito); Greece: K Douros, National and Kapodistrian University of Athens (Athens); Honduras: SM Sosa Ferrari, Instituto Nacional Cardiopulmonar (Tegucigalpa); India: M Sabir, Kothari Medical & Research Institute (Bikaner); M Singh, Postgraduate Institute of Medical Education and Research (Chandigarh); V Singh*, Asthma Bhawan (Jaipur); AG Ghoshal, National Allergy Asthma Bronchitis Institute (Kolkata); TU Sukumaran, Pushpagiri Institute of Medical Sciences and Research, Thiruvalla, Kottayam (Kottayam); S Awasthi, King George's Medical University (Lucknow); PA Mahesh, JSS Medical College, JSSAHER (Mysuru); SK Kabra, All India Institute of Medical Sciences (New Delhi (7)); S Salvi, Chest Research Foundation (Pune); Iran: M Tavakol, Alborz University of Medical Sciences (Karaj); N Behniafard, Shahid Sadoughi University of Medical Sciences (Yazd); Kingdom of Saudi Arabia: SA Alomary, Ministry of Health (Kingdom of Saudi Arabia); Kosovo: I Bucaliu‐Ismajli, The principal center of family care (Ferizaj); L Pajaziti, University Hospital Clinic, Clinic of Dermatology, Prishtina, Kosovo (Gjakova); V Gashi, American Hospital in Kosovo (Gjilan); X Kurhasani, UBT College Kosovo (Peja 13–14); B Gacaferri‐Lumezi, University of Prishtina Hasan Prishtina (Peja 6–7); LN Ahmetaj*, University Hospital (Prishtina); V Zhjeqi, University of Prishtina (Prizren); México: MG Sanchez Coronel, COMPEDIA (Colegio Mexicano de PediatrasEspecialistas en Inmunología y Alergia) (Aguascalientes); HL Moreno Gardea, Hospital Angeles Chihuahua (Chihuahua); G Ochoa‐Lopez, Department of Paediatric Allergology (Ciudad Juárez); R García‐Almaráz, Hospital Infantil de Tamaulipas (Ciudad Victoria); JA Sacre Hazouri, Instituto Privado de Alergia, (Córdoba); N Rodriguez‐Perez, Instituto de Ciencias y Estudios Superiores de Tamaulipas (Matamoros); JV Mérida‐Palacio, Centro de Investigacion de Enfermedades Alergicas y Respiratorias (Mexicali); BE Del Río Navarro*, Service of Allergy and Clinical Immunology, Hospital Infantil de México (México City (North Area)); LO Hernández‐Mondragón, CRIT de Michoacán (Michoacán); SN González‐Díaz, Universidad Autónoma de Nuevo León (Monterrey); R Garcia‐Muñoz, Universidad Regional del Sureste (Oaxaca); Md Juan Pineda, Universidad de Guadalajara (Puerto Vallarta); Bd Ramos García, Instituto Mexicano del Seguro Social (San Luis Potosí); AJ Escalante‐Dominguez, Hospital General Tijuana [Isesalud] (Tijuana); FJ Linares‐Zapién, Centro De Enfermedades Alergicas Y Asma de Toluca (Toluca Rural); EM Navarrete‐Rodriguez, Hospital Infantil de Mexico Federico Gomez (Toluca Urban Area); J Santos Lozano, Medica san Angel (Xalapa); New Zealand: I Asher, University of Auckland (Auckland); Nicaragua: JF Sánchez, Hospital Infantil Manuel de Jesús Rivera (Managua); Nigeria: AG Falade, University of Ibadan and University College Hospital (Ibadan); Poland: G Brożek, Medical University of Silesia (Katowice); Russia: K Kyzmicheva, Tyumen State Medical University (Tyumen); South Africa: HJ Zar, SA MRC Unit on Child & Adolescent Health (Cape Town); R Masekela, University of Kwazulu‐Natal (Durban); Spain: A López‐Silvarrey Varela, Fundacion Maria Jose Jove (A Coruña); C González Díaz, Universidad del País Vasco UPV/EHU (Bilbao); A Bercedo Sanz, Cantabrian Health Service, Valdecilla Research Institute (IDIVAL), Dobra Health Center, Torrelavega, Cantabria, Spain (Cantabria); L García‐Marcos*, Paediatric Allergy and Pulmonology Units, Virgen de la Arrixaca University Children‘s Hospital, University of Murcia and IMIB Bioresearch Institute, Murcia; (Cartagena); J Pellegrini Belinchon, Universidad de Salamanca (Salamanca); Sri Lanka: JC Ranasinghe, Teaching Hospital Peradeniya (Anuradhapura); ST Kudagammana, University of Peradeniya (Peradeniya); Sudan: H El Sadig, Ministry of Health (Gadarif); M Nour, Epi‐Lab (Khartoum); Syrian Arab Republic: G Alkhayer, Syrian Private University (Damascus); G Dib, National Center for research and training for chronic respiratory disease and co‐morbidities, Tishreen University (Lattakia 13–14); Y Mohammad*, National Center for research and training for chronic respiratory disease and co‐morbidities, Tishreen University (Lattakia 6–7); Taiwan: JL Huang, Department ofPaediatrics, Chang Gung Memorial Hospital, New Taipei Municipal TuChen Hospital, and Chang Gung University (Taipei); Thailand: S Chinratanapisit, Department ofPaediatrics, Bhumibol Adulyadej Hospital, Royal Thai Air Force (Bangkok).

* National Coordinators.

**Global Asthma Network National Co‐ordinators not named above:** Brazil: D Solé, Escola Paulista de Medicina, Federal University of São Paulo; Costa Rica: ME Soto‐Quirós, University of Costa Rica; Kingdom of Saudi Arabia: WA Althagafi, Ministry of Health, Kingdom of Saudi Arabia; Sudan: A El Sony, Epidemiological Laboratory (Epi‐Lab) for Public Health, Research and Development; Thailand: P Vichyanond, Mahidol University.
