# Supplementary material for: Worldwide Impact of Human Development and Inequality on the Prevalence of Asthma, Rhinoconjunctivitis and Eczema. Global Asthma Network's Ecological Study
Source: Clin Exp Allergy. 2026 Jun 4;56(9):1048–62. doi: 10.1111/cea.70355 (PMC13532778; doi:10.1111/cea.70355)
Supplement: Supplementary file 1 [file CEA-56-1048-s001.docx]

**IMPACT OF HUMAN DEVELOPMENT AND INEQUALITY ON THE PREVALENCE OF ASTHMA, RHINOCONJUNCTIVITIS AND ECZEMA: GLOBAL ASTHMA NETWORK’S ECOLOGICAL STUDY.**

**Supplementary material (web tables and figures).**

|  | Page |
| --- | --- |
| **Web table 1.** Impact of the different moderators on the variability (adjusted R^2^) of the prevalence of **current wheeze** between centres. Simple (crude) and multiple meta-regression analyses (full and best adjusted models). | 3 |
| **Web table 2.** Impact of the different moderators on the variability (adjusted R^2^) of the prevalence of **symptoms of severe asthma** between centres. Simple (crude) and multiple meta-regression analyses (full and best adjusted models). | 4 |
| **Web table 3.** Impact of the different moderators on the variability (adjusted R^2^) of the centre prevalence of **asthma ever**. Simple (crude) and multiple meta-regression analyses (full and best adjusted models). | 5 |
| **Web table 4.** Impact of the different moderators on the variability (adjusted R^2^) of the prevalence of **current rhinoconjunctivitis** symptoms between centres. Simple (crude) and multiple meta-regression analyses (full and best adjusted models). | 6 |
| **Web table 5.** Impact of the different moderators on the variability (adjusted R^2^) of the prevalence of **symptoms of severe rhinoconjunctivitis** between centres. Simple (crude) and multiple meta-regression analyses (full and best adjusted models). | 7 |
| **Web table 6.** Impact of the different moderators on the variability (adjusted R^2^) of the centre prevalence of **hay fever ever**. Simple (crude) and multiple meta-regression analyses (full and best adjusted models). | 8 |
| **Web table 7.** Impact of the different moderators on the variability (adjusted R^2^) of the prevalence of **current eczema symptoms** between centres. Simple (crude) and multiple meta-regression analyses (full and best adjusted models). | 9 |
| **Web table 8.** Impact of the different moderators on the variability (adjusted R^2^) of the prevalence of **symptoms of severe eczema** between centres. Simple (crude) and multiple meta-regression analyses (full and best adjusted models). | 10 |
| **Web table 9.** Impact of the different moderators on the variability (adjusted R^2^) of the prevalence of **eczema ever** between centres. Simple (crude) and multiple meta-regression analyses (full and best adjusted models). | 11 |
| **Web Figure 1.** Residual plots of “current wheeze”, “symptoms of severe asthma” and “asthma ever” in children, adolescents and adults after applying the best adjusted models of the meta-regressions analyses depicted in Web tables 1-3. | 12 |
| **Web Figure 2**. Residual plots of “current rhinoconjunctivitis symptoms” and “symptoms of severe rhinoconjunctivitis” in children and adolescents after applying the best adjusted models of the meta-regressions analyses depicted in Web tables 4-5. (Those two markers were not available in adults and no regression which included HDI and GinI could be fitted for the common one in the three age groups i.e. “hay fever”). | 13 |
| **Web Figure 3**. Residual plots of “current eczema symptoms” and “eczema ever” in children and adolescents after applying the best adjusted models of the meta-regressions analyses depicted in Web tables 7 and 9. (No regression which included HDI and GinI could be fitted for “symptoms of severe eczema” in those age groups and was not available in adults. GinI was not a significant covariate in the best adjusted model for “eczema ever” in adults). | 14 |
| **Web Figure 4**. Regression plots of “current wheeze”, “symptoms of severe asthma” and “asthma ever” in children, adolescents and adults after applying the best adjusted models of the meta-regressions analyses depicted in Web tables 1-3. | 15 |
| **Web Figure 5.** Regression plots of “current rhinoconjunctivitis symptoms” and “symptoms of severe rhinoconjunctivitis” in children and adolescents after applying the best adjusted models of the meta-regressions analyses depicted in Web tables 4-5. (Those two markers were not available in adults and no regression which included HDI and GinI could be fitted for the common one in the three age groups i.e. “hay fever”). | 16 |
| **Web Figure 6**. Regression plots of “current eczema symptoms” and “eczema ever” in children and adolescents after applying the best adjusted models of the meta-regressions analyses depicted in Web tables 7 and 9. (No regression which included HDI and GinI could be fitted for “symptoms of severe eczema” in those age groups and was not available in adults. GinI was not a significant covariate in the best adjusted model for “eczema ever” in adults). | 17 |
| **Web Figure 7**. Heatmap of areas of high and low prevalence of “current wheeze”, “symptoms of severe asthma” and “asthma ever” in children, adolescents and adults after applying the best adjusted models of the meta-regressions analyses to the range of values of HDI and GinI in the study. The prevalence range of the specific disease marker is the one of the fitted values. | 18 |
| **Web Figure 8.** Heatmap of areas of high and low prevalence of “current rhinoconjunctivitis symptoms” and “symptoms of severe rhinoconjunctivitis” in children and adolescents after applying the best adjusted models of the meta-regressions analyses to the range of values of HDI and GinI in the study. The prevalence range of the specific disease marker is the one of the fitted values. (Both “rhinoconjunctivitis” markers were not available in adults and no regression which included HDI and GinI could be fitted for the common one in the three age groups i.e. “hay fever”). | 19 |
| **Web Figure 9.** Heatmap of areas of high and low prevalence of “current eczema symptoms” and “eczema ever” in children and adolescents after applying the best adjusted models of the meta-regressions analyses to the range of values of HDI and GinI in the study. The prevalence range of the specific disease marker is the one of the fitted values. (No regression which included HDI and GinI could be fitted for “symptoms of severe eczema” in those age groups and was not available in adults. GinI was not a significant covariate in the best adjusted model for “eczema ever” in adults). | 20 |

Web table 1. Impact of the different moderators on the variability (adjusted R^2^) of the prevalence of **current wheeze** between centres. Simple (crude) and multiple meta-regression analyses (full and best adjusted models).

|  | Crude | | | |  | Fully adjusted model | | | | |  | Best adjusted model | | | | | |
| --- | --- | --- | --- | --- | --- | --- | --- | --- | --- | --- | --- | --- | --- | --- | --- | --- | --- |
|  | Exp  (95%CI) | tau^2^ | aR^2^  (%) | p |  | aExp  (95%CI) | Model  F | Model  aR^2^  (%) | Model  tau^2^ | Model  p |  | aExp  (95%CI) | Model  F | Model  aR^2^  (%) | Model  tau^2^ | Model  p | Contribution  to aR^2^  (%) |
| **CHILDREN** | | | | | | | | | | | | | | | | | |
| HDI | 5·6523  (3·2338;8·0707) | 0·3841 | 35·5 | <0·001 |  | 7·7084  (5·6756;9·7412) | 16·09 | 69·3 | 0·1831 | <0·001 |  | 7·7270  (5·7367;9·7174) | 20·58 | 70·1 | 0·1778 | <0·001 | 35·5 |
| GINI | 0·0410  (0·0023;0·0796) | 0·5469 | 8·2 | 0·038 |  | 0·0690  (0·0401;0·0979) |  |  |  |  |  | 0·0673  (0·0431;0·0914) |  |  |  |  | 21·6 |
| Humidity^†^ | 0·0098  (-0·0036;0·0233) | 0·5810 | 2·5 | 0·148 |  | 0·0125  (0·0033;0·0216) |  |  |  |  |  | 0·0120  (0·0039;0·0201) |  |  |  |  | 4·4 |
| Temperature^‡^ | -0·0137  (-0·0592;0·0318) | 0·6075 | -2·01 | 0·547 |  | 0·0472  (0·0096;0·0848) |  |  |  |  |  | 0·0448  (0·0154;0·0742) |  |  |  |  | 8·6 |
| Latitude^¥^ | -0·0067  (-0·0286;0·0151) | 0·6052 | -1·61 | 0·536 |  | 0·0020  (-0·0174;0·0215) |  |  |  |  |  | N·I· |  |  |  |  |  |
| **ADOLESCENTS** | | | | | | | | | | | | | | | | | |
| HDI | 2·5053  (0·7243;4·2863) | 0·3972 | 10·50 | 0·007 |  | 2·1677  (0·2802;4·0550) | 5·34 | 27·4 | 0·3223 | <0·001 |  | 2·6857  (1·0779;4·2935) | 12·26 | 28·0 | 0·3194 | <0·001 | 10·5 |
| GINI | 0·0339  (0·0139;0·0539) | 0·3767 | 15·13 | 0·001 |  | 0·0398  (0·0197;0·0600) |  |  |  |  |  | 0·0357  (0·0171;0·0542) |  |  |  |  | 17·5 |
| Humidity^†^ | 0·0072  (-0·0029;0·0172) | 0·4363 | 1·68 | 0·161 |  | 0·0062  (-0·0034;0·0158) |  |  |  |  |  | N·I· |  |  |  |  |  |
| Temperature^‡^ | -0·0200  (-0·0491;0·0092) | 0·4337 | 1·37 | 0·176 |  | 0·0031  (-0·0330;0·0392) |  |  |  |  |  | N·I· |  |  |  |  |  |
| Latitude^¥^ | 0·0039  (-0·0105;0·0183) | 0·449 | -1·18 | 0·589 |  | 0·0081  (-0·0095;0·0257) |  |  |  |  |  | N·I· |  |  |  |  |  |
| **ADULTS** | | | | | | | | | | | | | | | | | |
| HDI | 3·5451  (1·0254;6·0649) | 0·4335 | 15·0 | 0·007 |  | 3·0653  (0·7121;5·4185) | 6·11 | 38·7 | 0·3127 | <0·001 |  | 3·4057  (1·2940;5·5174) | 10·32 | 40·9 | 0·3012 | <0·001 | 15·0 |
| GINI | 0·0474  (0·0204;0·0745) | 0·3986 | 21·8 | 0·001 |  | 0·0527  (0·0235;0·0820) |  |  |  |  |  | 0·0511  (0·0270;0·0754) |  |  |  |  | 21·9 |
| Humidity^†^ | 0·0058  (-0·0077;0·0192) | 0·5132 | -0·62 | 0·390 |  | 0·0089  (-0·0033;0·0210) |  |  |  |  |  | 0·0101  (-0·0005;0·0207) |  |  |  |  | 4·0 |
| Temperature^‡^ | -0·0188  (-0·0559;0·0182) | 0·509 | 0·19 | 0·310 |  | -0·0147  (-0·0578;0·0285) |  |  |  |  |  | N·I· |  |  |  |  |  |
| Latitude^¥^ | -0·0065  (-0·0263;0·0133) | 0·5172 | -1·41 | 0·509 |  | -0·0013  (-0·0259;0·0232) |  |  |  |  |  | N·I· |  |  |  |  |  |

CHILDREN: Summary prevalence* (44 centres): 7·9% (95%CI: 6·4;9·8), tau2=0·5956, Q=1939·18 p<0·001, I2=99·10%)

ADOLESCENTS: Summary prevalence* (63 centres): 9·9% (95%CI: 8·5;11·5), tau2=0·4438, Q=3267·11 p<0·001, I2=99·06%)

ADULTS: Summary prevalence* (43 centres): 7·0% (95%CI: 5·7;8·6), tau2=0·5100, Q=4826·83 p<0·001, I2=99·27%)

HDI: Human development index (0-1). GINI: Gini index of inequality (0-100). NI: Not included in the model.

*Random effects metanalysis (restricted maximum likelihood method). ^†^Mean annual relative humidity (%). ^‡^Mean annual temperature (°C). ^¥^Absolute distance from Equator.

Web Table 2. Impact of the different moderators on the variability (adjusted R^2^) of the prevalence of **symptoms of severe asthma** between centres. Simple (crude) and multiple meta-regression analyses (full and best adjusted models).

|  | Crude | | | |  | Fully adjusted model | | | | |  | Best adjusted model | | | | | |
| --- | --- | --- | --- | --- | --- | --- | --- | --- | --- | --- | --- | --- | --- | --- | --- | --- | --- |
|  | Exp  (95%CI) | tau^2^ | aR^2^  (%) | p |  | aExp  (95%CI) | Model  F | Model  aR^2^  (%) | Model  tau^2^ | Model  p |  | aExp  (95%CI) | Model  F | Model  aR^2^  (%) | Model  tau^2^ | Model  p | Contribution  to aR^2^  (%) |
| **CHILDREN** | | | | | | | | | | | | | | | | | |
| HDI | 4·7450  (1·8442;7·6456) | 0·4743 | 19·3 | 0·002 |  | 7·1865  (4·9673;9·4056) | 14·62 | 69·7 | 0·1782 | <0·001 |  | 7·2590  (5·0835;9·4344) | 18·54 | 70·2 | 0·1753 | <0·001 | 19·3 |
| GINI | 0·0595  (0·0208;0·0982) | 0·4665 | 20·7 | 0·003 |  | 0·0923  (0·0613;0·1232) |  |  |  |  |  | 0·0880  (0·0619;0·1141) |  |  |  |  | 36·8 |
| Humidity^†^ | 0·0088  (-0·0058;0·0234) | 0·5896 | -0·27 | 0·232 |  | 0·0144  (0·0047;0·0241) |  |  |  |  |  | 0·0133  (0·0047;0·0219) |  |  |  |  | 5·2 |
| Temperature^‡^ | -0·0086  (-0·0579;0·0408) | 0·6079 | -3·38 | 0·728 |  | 0·0504  (0·0107;0·0901) |  |  |  |  |  | 0·0442  (0·0132;0·0752) |  |  |  |  | 8·9 |
| Latitude^¥^ | -0·0112  (-0·0344;0·0120) | 0·5872 | 0·14 | 0·335 |  | 0·0053  (-0·0152;0·0258) |  |  |  |  |  | N·I· |  |  |  |  |  |
| **ADOLESCENTS** | | | | | | | | | | | | | | | | | |
| HDI | 1·1604  (-0·8467;3·1675) | 0·499 | 0·48 | 0·252 |  | 0·8092  (-1·2887;2·9070) | 4·20 | 21·8 | 0·3923 | 0·0025 |  | 1·3789  (-0·4096;3·1674) | 9·17 | 22·0 | 0·391 | <0·001 | 0·5 |
| GINI | 0·0412  (0·0203;0·0620) | 0·4003 | 20·16 | <0·001 |  | 0·0463  (0·0239;0·0686) |  |  |  |  |  | 0·0421  (0·0215;0·0628) |  |  |  |  | 21·5 |
| Humidity^†^ | 0·0073  (-0·0035;0·0181) | 0·4943 | 1·42 | 0·182 |  | 0·0078  (-0·0028;0·0184) |  |  |  |  |  | N·I· |  |  |  |  |  |
| Temperature^‡^ | -0·0096  (-0·0413;0·0221) | 0·5072 | -1·16 | 0·547 |  | 0·0031  (-0·0371;0·0432) |  |  |  |  |  | N·I· |  |  |  |  |  |
| Latitude^¥^ | -0·0019  (-0·0174;0·0136) | 0·5099 | -1·70 | 0·810 |  | 0·0080  (-0·0115;0·0275) |  |  |  |  |  | N·I· |  |  |  |  |  |
| **ADULTS** | | | | | | | | | | | | | | | | | |
| HDI | 3·8665  (0·8523;6·8807) | 0·6095 | 12·56 | 0·013 |  | 3·2891  (0·6860;5·8922) | 7·86 | 46·7 | 0·3716 | <0·001 |  | 3·6701  (1·3221;6·0181) | 13·08 | 48·3 | 0·3605 | <0·001 | 12·6 |
| GINI | 0·0644  (0·0342;0·0947) | 0·4828 | 30·75 | <0·001 |  | 0·0688  (0·0364;0·1012) |  |  |  |  |  | 0·0694  (0·0424;0·0964) |  |  |  |  | 30·9 |
| Humidity^†^ | 0·0067  (-0·0091;0·0226) | 0·7025 | -0·78 | 0·398 |  | 0·0102  (-0·0032;0·0236) |  |  |  |  |  | 0·0125  (0·0007;0·0243) |  |  |  |  | 4·8 |
| Temperature^‡^ | -0·0200  (-0·0639;0·0239) | 0·6995 | -0·34 | 0·363 |  | -0·0233  (-0·071;0·0244) |  |  |  |  |  | N·I· |  |  |  |  |  |
| Latitude^¥^ | -0·0124  (-0·0356;0·0107) | 0·6946 | 0·36 | 0·285 |  | -0·0057  (-0·0329;0·0214) |  |  |  |  |  | N·I· |  |  |  |  |  |

CHILDREN: Summary prevalence* (44 centres): 3·3% (95%CI: 2·6;4·1), tau2=0·5880, Q=1162·82 p<0·001, I2=98·04%)

ADOLESCENTS: Summary prevalence* (63 centres): 4·5% (95%CI: 3·8;5·3), tau2=0·5014, Q=2318·13 p<0·001, I2=98·36%)

ADULTS: Summary prevalence* (43 centres): 2·7% (95%CI: 2·1;3·4), tau2=0·6971, Q=2177·39 p<0·001, I2=98·7%)

HDI: Human development index (0-1). GINI: Gini index of inequality (0-100). NI: Not included in the model.

*Random effects metanalysis (restricted maximum likelihood method). ^†^Mean annual relative humidity (%). ^‡^Mean annual temperature (°C). ^¥^Absolute distance from Equator.

Web table 3. Impact of the different moderators on the variability (adjusted R^2^) of the centre prevalence of **asthma ever**. Simple (crude) and multiple meta-regression analyses (full and best adjusted models).

|  | Crude | | | |  | Fully adjusted model | | | | |  | Best adjusted model | | | | | |
| --- | --- | --- | --- | --- | --- | --- | --- | --- | --- | --- | --- | --- | --- | --- | --- | --- | --- |
|  | Exp  (95%CI) | tau^2^ | aR^2^  (%) | p |  | aExp  (95%CI) | Model  F | Model  aR^2^  (%) | Model  tau^2^ | Model  p |  | aExp  (95%CI) | Model  F | Model  aR^2^  (%) | Model  tau^2^ | Model  p | Contribution  to aR^2^  (%) |
| **CHILDREN** | | | | | | | | | | | | | | | | | |
| HDI | 8·4402  (5·4986;11·3817) | 0·5845 | 45·0 | <0·001 |  | 9·8572  (6·7485;12·9658) | 10·86 | 55·60 | 0·4718 | <0·001 |  | 9·0712  (6·3449;11·7975) | 17·84 | 55·9 | 0·4682 | <0·001 | 45·0 |
| GINI | 0·0242  (-0·0290;0·0774) | 1·07 | -0·66 | 0·364 |  | 0·0621  (0·0175;0·1067) |  |  |  |  |  | 0·0585  (0·0213;0·0957) |  |  |  |  | 7·6 |
| Humidity^†^ | 0·0139  (-0·0037;0·0316) | 1·026 | 3·49 | 0·119 |  | 0·0141  (-0·0001;0·0283) |  |  |  |  |  | 0·0122  (-0·0001;0·0245) |  |  |  |  | 3·3 |
| Temperature^‡^ | -0·0478  (-0·1059;0·0104) | 1·023 | 3·69 | 0·105 |  | 0·0310  (-0·0276;0·0888) |  |  |  |  |  | N·I· |  |  |  |  |  |
| Latitude^¥^ | 0·0064  (-0·0224;0·0353) | 1·083 | -1·95 | 0·656 |  | 0·0030  (-0·0272;0·0332) |  |  |  |  |  | N·I· |  |  |  |  |  |
| **ADOLESCENTS** | | | | | | | | | | | | | | | | | |
| HDI | 3·2902  (0·9670;5·6134) | 0·6679 | 11·21 | 0·006 |  | 4·3202  (1·7190;6·9214) | 3·70 | 19·63 | 0·6045 | 0·0057 |  | 4·4318  (1·9197;6·9439) | 6·16 | 21·9 | 0·5877 | 0·0010 | 11·2 |
| GINI | 0·0312  (0·0038;0·0587) | 0·704 | 6·41 | 0·027 |  | 0·0316  (0·0037;0·0594) |  |  |  |  |  | 0·0303  (0·0047;0·0560) |  |  |  |  | 8·0 |
| Humidity^†^ | 0·0047  (-0·0087;0·0180) | 0·7591 | -0·92 | 0·487 |  | 0·0046  (-0·0086;0·0178) |  |  |  |  |  | N·I· |  |  |  |  |  |
| Temperature^‡^ | 0·0039  (-0·0349;0·0427) | 0·7648 | -1·68 | 0·841 |  | 0·0380  (-0·0118;0·0880) |  |  |  |  |  | 0·0316  (-0·0082;0·0715) |  |  |  |  | 2·7 |
| Latitude^¥^ | -0·0028  (-0·0218;0·0161) | 0·7647 | -1·67 | 0·767 |  | 0·0031  (-0·0212;0·0274) |  |  |  |  |  | N·I· |  |  |  |  |  |
| **ADULTS** | | | | | | | | | | | | | | | | | |
| HDI | 5·7711  (2·5104;9·0318) | 0·7247 | 22·66 | 0·001 |  | 7·1249  (4·2921;9·9578) | 9·75 | 51·67 | 0·4529 | <0·001 |  | 7·0801  (4·3099;9·8503) | 12·48 | 52·9 | 0·4413 | <0·001 | 22·7 |
| GINI | 0·626  (0·0254;0·0999) | 0·7513 | 19·82 | 0·002 |  | 0·0551  (0·0197;0·0904) |  |  |  |  |  | 0·0572  (0·0270;0·0874) |  |  |  |  | 19·6 |
| Humidity^†^ | 0·0058  (-0·0125;0·0241) | 0·9503 | -1·42 | 0·526 |  | 0·0144  (-0·0002;0·0290) |  |  |  |  |  | 0·0151  (0·0020;0·0283) |  |  |  |  | 2·4 |
| Temperature^‡^ | 0·0257  (-0·0247;0·0761) | 0·9379 | -0·09 | 0·310 |  | 0·0509  (-0·0012;0·1030) |  |  |  |  |  | 0·0549  (0·0149;0·0948) |  |  |  |  | 8·2 |
| Latitude^¥^ | -0·0237  (-0·0497;0·0024) | 0·8898 | 5·04 | 0·074 |  | -0·0036  (-0·0332;0·0261) |  |  |  |  |  | N·I· |  |  |  |  |  |

CHILDREN: Summary prevalence* (44 centres): 5·4% (95%CI: 4·0;7·2), tau2=1·0627, Q=3860·78 p<0·001, I2=99·38%)

ADOLESCENTS: Summary prevalence* (63 centres): 8·3% (95%CI: 6·8;10·1), tau2=0·7522, Q=5639·63 p<0·001, I2=99·4%)

ADULTS: Summary prevalence* (43 centres): 3·6% (95%CI: 2·7;4·8), tau2=0·9370, Q=7306·15 p<0·001, I2=99·4%)

HDI: Human development index (0-1). GINI: Gini index of inequality (0-100). NI: Not included in the model.

*Random effects metanalysis (restricted maximum likelihood method). ^†^Mean annual relative humidity (%). ^‡^Mean annual temperature (°C). ^¥^Absolute distance from Equator.

Web table 4. Impact of the different moderators on the variability (adjusted R^2^) of the prevalence of **current rhinoconjunctivitis** symptoms between centres. Simple (crude) and multiple meta-regression analyses (full and best adjusted models).

|  | Crude | | | |  | Fully adjusted model | | | | |  | Best adjusted model | | | | | |
| --- | --- | --- | --- | --- | --- | --- | --- | --- | --- | --- | --- | --- | --- | --- | --- | --- | --- |
|  | Exp  (95%CI) | tau^2^ | aR^2^  (%) | p |  | aExp  (95%CI) | Model  F | Model  aR^2^  (%) | Model  tau^2^ | Model  p |  | aExp  (95%CI) | Model  F | Model  aR^2^  (%) | Model  tau^2^ | Model  p | Contribution  to aR^2^  (%) |
| **CHILDREN** | | | | | | | | | | | | | | | | | |
| HDI | 6·3109  (3·4131;9·2087) | 0·5359 | 32·02 | <0·001 |  | 7·7184  (4·8350;10·6019) | 8·91 | 50·9 | 0·3895 | <0·001 |  | 7·2349  (4·7254;9·7444) | 15·17 | 52·7 | 0·3752 | <0·001 | 32·0 |
| GINI | 0·0442  (-0·0013;0·0897) | 0·7412 | 6·6 | 0·056 |  | 0·0725  (0·0315;0·1134) |  |  |  |  |  | 0·0719  (0·0379;0·1058) |  |  |  |  | 18·7 |
| Humidity^†^ | 0·0096  (-0·0063;0·0255) | 0·7897 | 0·49 | 0·232 |  | 0·0105  (-0·0025;0·0235) |  |  |  |  |  | 0·0098  (-0·0015;0·0211) |  |  |  |  | 2·0 |
| Temperature^‡^ | -0·0404  (-0·0922;0·0114) | 0·7716 | 2·76 | 0·123 |  | 0·0151  (-0·0382;0·0683) |  |  |  |  |  | N·I· |  |  |  |  |  |
| Latitude^¥^ | -0·0005  (-0·0261;0·0252) | 0·8157 | -2·79 | 0·971 |  | -0·0003  (-0·0278;0·0272) |  |  |  |  |  | N·I· |  |  |  |  |  |
| **ADOLESCENTS** | | | | | | | | | | | | | | | | | |
| HDI | 1·7819  (0·5604;3·0034) | 0·1872 | 11·2 | 0·005 |  | 1·5555  (0·1723;2·9387) | 3·44 | 17·0 | 0·1749 | 0·009 |  | 1·8564  (0·6897;3·023) | 8·18 | 19·4 | 0·1698 | <0·001 | 11·2 |
| GINI | 0·0168  (0·0023;0·0313) | 0·1965 | 6·8 | 0·024 |  | 0·0205  (0·0058;0·0353) |  |  |  |  |  | 0·0178  (0·0043;0·0313) |  |  |  |  | 8·2 |
| Humidity^†^ | 0·0037  (-0·0033;0·0107) | 0·2103 | 0·2 | 0·297 |  | 0·0028  (-0·0042;0·0099) |  |  |  |  |  | N·I· |  |  |  |  |  |
| Temperature^‡^ | -0·0143  (-0·0342;0·0056) | 0·2071 | 1·8 | 0·157 |  | 0·0023  (-0·0240;0·0287) |  |  |  |  |  | N·I· |  |  |  |  |  |
| Latitude^¥^ | 0·0046  (-0·0053;0·0146) | 0·2112 | -0·21 | 0·354 |  | 0·0055  (-0·0073;0·0184) |  |  |  |  |  | N·I· |  |  |  |  |  |

CHILDREN: Summary prevalence* (44 centres): 6·0% (95%CI: 4·7;7·7), tau2=0·7936, Q=3046·54 p<0·001, I2=99·20%)

ADOLESCENTS: Summary prevalence* (62 centres): 12·8% (95%CI: 11·6;14·2), tau2=0·2108, Q=3227·49 p<0·001, I2=98·4%)

HDI: Human development index (0-1). GINI: Gini index of inequality (0-100). NI: Not included in the model.

*Random effects metanalysis (restricted maximum likelihood method). ^†^Mean annual relative humidity (%). ^‡^Mean annual temperature (°C). ^¥^Absolute distance from Equator.

Web table 5. Impact of the different moderators on the variability (adjusted R^2^) of the prevalence of **symptoms of severe rhinoconjunctivitis** between centres. Simple (crude) and multiple meta-regression analyses (full and best adjusted models).

|  | Crude | | | |  | Fully adjusted model | | | | |  | Best adjusted model | | | | | |
| --- | --- | --- | --- | --- | --- | --- | --- | --- | --- | --- | --- | --- | --- | --- | --- | --- | --- |
|  | Exp  (95%CI) | tau^2^ | aR^2^  (%) | p |  | aExp  (95%CI) | Model  F | Model  aR^2^  (%) | Model  tau^2^ | Model  p |  | aExp  (95%CI) | Model  F | Model  aR^2^  (%) | Model  tau^2^ | Model  p | Contribution  to aR^2^  (%) |
| **CHILDREN** | | | | | | | | | | | | | | | | | |
| HDI | 2·9396  (-0·1414;6·0206) | 0·4012 | 1·3 | 0·061 |  | 5·3320  (1·9494;8·7146) | 3·19 | 16·1 | 0·3411 | 0·0174 |  | 4·6008  (1·5132;7·6885) | 6·59 | 17·3 | 0·3363 | 0·0034 | 1·3 |
| GINI | 0·0357  (-0·0023;0·0737) | 0·3868 | 4·9 | 0·065 |  | 0·0680  (0·0210;0·1150) |  |  |  |  |  | 0·0566  (0·0188;0·0945) |  |  |  |  | 16·0 |
| Humidity^†^ | 0·0031  (-0·0107;0·0168) | 0·4242 | -4·3 | 0·654 |  | 0·0100  (-0·0048;0·0239) |  |  |  |  |  | N·I· |  |  |  |  |  |
| Temperature^‡^ | -0·0009  (-0·0464;0·0445) | 0·4224 | -3·9 | 0·968 |  | 0·0276  (-0·0294;0·0847) |  |  |  |  |  | N·I· |  |  |  |  |  |
| Latitude^¥^ | -0·0066  (-0·0278;0·0145) | 0·4104 | -0·95 | 0·530 |  | 0·0032  (-0·0268;0·0331) |  |  |  |  |  | N·I· |  |  |  |  |  |
| **ADOLESCENTS** | | | | | | | | | | | | | | | | | |
| HDI | 1·4109  (-0·6686;3·4904) | 0·4393 | 1·4 | 0·180 |  | 1·7255  (-0·4534;3·9044) | 4·17 | 28·0 | 0·3205 | 0·0028 |  | 1·7255  (-0·4534;3·9044) | 4·17 | 28·0 | 0·3205 | 0·0028 | 1·4 |
| GINI | 0·0284  (0·0058;0·0509) | 0·4017 | 9·8 | 0·015 |  | 0·0361  (0·0139;0·0582) |  |  |  |  |  | 0·0361  (0·0139;0·0582) |  |  |  |  | 11·8 |
| Humidity^†^ | 0·0093  (-0·0019;0·0204) | 0·4315 | 3·1 | 0·101 |  | 0·0136  (0·0028;0·0244) |  |  |  |  |  | 0·0136  (0·0028;0·0244) |  |  |  |  | 2·5 |
| Temperature^‡^ | 0·0083  (-0·0245;0·0411) | 0·4509 | -1·3 | 0·615 |  | 0·0534  (0·0121;0·0947) |  |  |  |  |  | 0·0534  (0·0121;0·0947) |  |  |  |  | 3·8 |
| Latitude^¥^ | 0·0028  (-0·0134;0·0190) | 0·4525 | -1·6 | 0·731 |  | 0·0219  (0·0020;0·0417) |  |  |  |  |  | 0·0219  (0·0020;0·0417) |  |  |  |  | 8·5 |

CHILDREN: Summary prevalence* (42 centres): 0·5% (95%CI: 0·4;0·6), tau2=0·4065, Q=288·08 p<0·001, I2=84·5%)

ADOLESCENTS: Summary prevalence* (61 centres): 0·70% (95%CI: 0·59;0·84), tau2=0·4454, Q=668·37 p<0·001, I2=90·3%)

HDI: Human development index (0-1). GINI: Gini index of inequality (0-100). NI: Not included in the model.

*Random effects metanalysis (restricted maximum likelihood method). ^†^Mean annual relative humidity (%). ^‡^Mean annual temperature (°C). ^¥^Absolute distance from Equator.

Web table 6. Impact of the different moderators on the variability (adjusted R2) of the centre prevalence of **hay fever ever**. Simple (crude) and multiple meta-regression analyses (full and best adjusted models).

|  | Crude | | | |  | Fully adjusted model | | | | |  | Best adjusted model | | | | | |
| --- | --- | --- | --- | --- | --- | --- | --- | --- | --- | --- | --- | --- | --- | --- | --- | --- | --- |
|  | Exp  (95%CI) | tau^2^ | aR^2^  (%) | p |  | aExp  (95%CI) | Model  F | Model  aR^2^  (%) | Model  tau^2^ | Model  p |  | aExp  (95%CI) | Model  F | Model  aR^2^  (%) | Model  tau^2^ | Model  p | Contribution  to aR^2^  (%) |
| **CHILDREN** | | | | | | | | | | | | | | | | | |
| HDI | 3·0858  (0·1125;6·0590) | 0·615 | 7·42 | 0·042 |  | 3·7666  (0·2531;7·2802) | 1·32 | 3·6 | 0·6404 | 0·2762 |  | Not consistent | | | | | |
| GINI | -0·0125  (-0·0548;0·0299) | 0·6745 | -1·54 | 0·556 |  | 0·0005  (-0·0503;0·0512) |  |  |  |  |  |  |  |  |  |  |  |
| Humidity^†^ | 0·0090  (-0·0050;0·0230) | 0·6544 | 1·50 | 0·203 |  | 0·0083  (-0·0078;0·0245) |  |  |  |  |  |  |  |  |  |  |  |
| Temperature^‡^ | -0·0023  (-0·0502;0·0456) | 0·681 | -2·51 | 0·924 |  | 0·0285  (-0·0381;0·0952) |  |  |  |  |  |  |  |  |  |  |  |
| Latitude^¥^ | -0·0002  (-0·0233;0·0230) | 0·6811 | -2·52 | 0·989 |  | -0·0014  (-0·0354;0·0325) |  |  |  |  |  |  |  |  |  |  |  |
| **ADOLESCENTS** | | | | | | | | | | | | | | | | | |
| HDI | 0·6488  (-1·7002;2·9978) | 0·7088 | -1·17 | 0·583 |  | 1·2748  (-1·3660;3·9156) | 1·79 | 6·11 | 0·6578 | 0·1296 |  | Not consistent | | | | | |
| GINI | -0·0060  (-0·0332;0·0211) | 0·7101 | -1·35 | 0·658 |  | 0·0003  (-0·0280;0·0286) |  |  |  |  |  |  |  |  |  |  |  |
| Humidity^†^ | 0·0035  (-0·0092;0·0162) | 0·7087 | -1·16 | 0·580 |  | 0·0100  (-0·0035;0·0235) |  |  |  |  |  |  |  |  |  |  |  |
| Temperature^‡^ | 0·0241  (-0·0125;0·0606) | 0·6924 | 1·18 | 0·193 |  | 0·0717  (0·0209;0·1225) |  |  |  |  |  |  |  |  |  |  |  |
| Latitude^¥^ | 0·0062  (-0·0119;0·0243) | 0·7069 | -0·89 | 0·499 |  | 0·0240  (-0·0007;0·0487) |  |  |  |  |  |  |  |  |  |  |  |
| **ADULTS** | | | | | | | | | | | | | | | | | |
| HDI | 5·2349  (1·9174;8·5524) | 0·7622 | 18·1 | 0·003 |  | 6·3803  (3·0013;9·7594) | 4·45 | 29·7 | 0·655 | 0·0028 |  | 6·3058  (2·8850;9·7267) | 5·66 | 25·3 | 0·6954 | 0·0026 | 18·1 |
| GINI | -0·0011  (-0·0427;0·0405) | 0·9538 | -2·4 | 0·957 |  | 0·0100  (-0·0319;0·0520) |  |  |  |  |  | N·I· |  |  |  |  |  |
| Humidity^†^ | 0·0073  (-0·0108;0·0254) | 0·9382 | -0·8 | 0·419 |  | 0·0178  (0·0003;0·0352) |  |  |  |  |  | N·I· |  |  |  |  |  |
| Temperature^‡^ | 0·0142  (-0·0360;0·0643) | 0·9465 | -1·7 | 0·572 |  | 0·0949  (0·0330;0·1568) |  |  |  |  |  | 0·0926  (0·0321;0·1531) |  |  |  |  | 5·7 |
| Latitude^¥^ | 0·0093  (-0·0173;0·0359) | 0·9422 | -1·20 | 0·486 |  | 0·0331  (-0·0021;0·0683) |  |  |  |  |  | 0·0290  (-0·0013;0·0592) |  |  |  |  | 1·5 |

CHILDREN: Summary prevalence* (43 centres): 9·9% (95%CI: 7·9;12·3), tau2=0·6643, Q=6713·79 p<0·001, I2=99·3%)

ADOLESCENTS: Summary prevalence* (62 centres): 13·7% (95%CI: 11·4;16·3), tau2=0·7006, Q=10974·01 p<0·001, I2=99·5%)

ADULTS: Summary prevalence* (43 centres): 11·7% (95%CI: 9·0;15·0), tau2=0·9310, Q=19252·59 p<0·001, I2=99·8%)

HDI: Human development index (0-1). GINI: Gini index of inequality (0-100). NI: Not included in the model.

*Random effects metanalysis (restricted maximum likelihood method). ^†^Mean annual relative humidity (%). ^‡^Mean annual temperature (°C). ^¥^Absolute distance from Equator.

Web table 7. Impact of the different moderators on the variability (adjusted R^2^) of the prevalence of **current eczema symptoms** between centres. Simple (crude) and multiple meta-regression analyses (full and best adjusted models).

|  | Crude | | | |  | Fully adjusted model | | | | |  | Best adjusted model | | | | | |
| --- | --- | --- | --- | --- | --- | --- | --- | --- | --- | --- | --- | --- | --- | --- | --- | --- | --- |
|  | Exp  (95%CI) | tau^2^ | aR^2^  (%) | p |  | aExp  (95%CI) | Model  F | Model  aR^2^  (%) | Model  tau^2^ | Model  p |  | aExp  (95%CI) | Model  F | Model  aR^2^  (%) | Model  tau^2^ | Model  p | Contribution  to aR^2^  (%) |
| **CHILDREN** | | | | | | | | | | | | | | | | | |
| HDI | 5·7701  (3·9449;7·5952) | 0·213 | 52·0 | <0·001 |  | 6·3819  (4·4251;8·3386) | 12·10 | 60·1 | 0·1767 | <0·001 |  | 5·9333  (4·1930;7·6736) | 19·0 | 59·2 | 0·1811 | <0·001 | 52·0 |
| GINI | 0·0028  (-0·0324;0·0380) | 0·4551 | -2·7 | 0·875 |  | 0·0342  (0·0062;0·0623) |  |  |  |  |  | 0·0260  (0·0021;0·0498) |  |  |  |  | 1·6 |
| Humidity^†^ | 0·0118  (0·0005;0·0231) | 0·4083 | 7·9 | 0·041 |  | 0·0125  (0·0036;0·0214) |  |  |  |  |  | 0·0098  (0·0019;0·0177) |  |  |  |  | 5·6 |
| Temperature^‡^ | -0·0338  (-0·0716;0·0040) | 0·4212 | 5·0 | 0·079 |  | 0·0291  (-0·0073;0·0656) |  |  |  |  |  | N·I |  |  |  |  |  |
| Latitude^¥^ | 0·0099  (-0·0088;0·0286) | 0·4408 | 0·6 | 0·292 |  | 0·0093  (-0·0095;0·0280) |  |  |  |  |  | N·I· |  |  |  |  |  |
| **ADOLESCENTS** | | | | | | | | | | | | | | | | | |
| HDI | 2·0874  (0·5496;3·6252) | 0·2978 | 9·8 | 0·009 |  | 1·5988  (-0·1899;3·3874) | 2·54 | 11·4 | 0·2923 | 0·0382 |  | 1·6220  (-0·0447;3·2888) | 3·75 | 12·1 | 0·2902 | 0·0156 | 9·8 |
| GINI | 0·0087  (-0·0100;0·0274) | 0·3312 | -0·3 | 0·354 |  | 0·0168  (-0·0023;0·0359) |  |  |  |  |  | 0·0155  (-0·0034;0·0344) |  |  |  |  | 0·4 |
| Humidity^†^ | 0·0051  (-0·0036;0·0139) | 0·3275 | 0·8 | 0·245 |  | 0·0054  (-0·0037;0·0145) |  |  |  |  |  | N·I· |  |  |  |  |  |
| Temperature^‡^ | -0·0193  (-0·0443;0·0058) | 0·3223 | 2·4 | 0·129 |  | 0·0123  (-0·0219;0·0465) |  |  |  |  |  | N·I· |  |  |  |  |  |
| Latitude^¥^ | 0·0118  (-0·0004;0·0239) | 0·3151 | 4·5 | 0·057 |  | 0·0148  (-0·0019;0·0314) |  |  |  |  |  | 0·0104  (-0·0032;0·0241) |  |  |  |  | 1·9 |

CHILDREN: Summary prevalence* (44 centres): 4·9% (95%CI: 4·1;6·0), tau2=0·4434, Q=1852·68 p<0·001, I2=98·2%)

ADOLESCENTS: Summary prevalence* (63 centres): 5·5% (95%CI: 4·8;6·3), tau2=0·3300, Q=3126·75 p<0·001, I2=98·0%)

HDI: Human development index (0-1). GINI: Gini index of inequality (0-100). NI: Not included in the model.

*Random effects metanalysis (restricted maximum likelihood method). ^†^Mean annual relative humidity (%). ^‡^Mean annual temperature (°C). ^¥^Absolute distance from Equator.

Web table 8. Impact of the different moderators on the variability (adjusted R^2^) of the prevalence of **symptoms of severe eczema** between centres. Simple (crude) and multiple meta-regression analyses (full and best adjusted models).

|  | Crude | | | |  | Fully adjusted model | | | | |  | Best adjusted model | | | | | |
| --- | --- | --- | --- | --- | --- | --- | --- | --- | --- | --- | --- | --- | --- | --- | --- | --- | --- |
|  | Exp  (95%CI) | tau^2^ | aR^2^  (%) | p |  | aExp  (95%CI) | Model  F | Model  aR^2^  (%) | Model  tau^2^ | Model  p |  | aExp  (95%CI) | Model  F | Model  aR^2^  (%) | Model  tau^2^ | Model  p | Contribution  to aR^2^  (%) |
| **CHILDREN** | | | | | | | | | | | | | | | | | |
| HDI | 3·4663  (1·0655;5·8672) | 0·262 | 23·0 | 0·006 |  | 4·7497  (2·3791;7·1204) | 7·0 | 61·3 | 0·1316 | <0·001 |  | 3·9548  (1·3896;6·5200) | 5·36 | 42·9 | 0·1954 | 0·004 | 23·0 |
| GINI | 0·0032  (-0·0326;0·0390) | 0·3545 | -4·2 | 0·857 |  | 0·05650  (0·0220;0·0910) |  |  |  |  |  | N·I· |  |  |  |  |  |
| Humidity^†^ | 0·0130  (0·0011;0·0249) | 0·285 | 16·3 | 0·033 |  | 0·0206  (0·0101;0·0312) |  |  |  |  |  | 0·0124  (0·0014;0·0234) |  |  |  |  | 11·4 |
| Temperature^‡^ | -0·0114  (-0·0516;0·0287) | 0·3518 | -3·4 | 0·568 |  | 0·0723  (0·0292;0·1155) |  |  |  |  |  | 0·0276  (-0·0114;0·0667) |  |  |  |  | 8·5 |
| Latitude^¥^ | 0·0127  (-0·0062;0·0317) | 0·3369 | 1·0 | 0·182 |  | 0·0311  (0·0090;0·0532) |  |  |  |  |  | N·I· |  |  |  |  |  |
| **ADOLESCENTS** | | | | | | | | | | | | | | | | | |
| HDI | 0·4512  (-1·4772;2·3796) | 0·3922 | -1·9 | 0·641 |  | -0·1860  (-2·2726;1·9005) | 3·22 | 18·3 | 0·3147 | 0·0130 |  | N·I· | 4·08 | 20·1 | 0·3078 | 0·0057 |  |
| GINI | 0·0256  (0·0045;0·0468) | 0·3484 | 9·5 | 0·018 |  | 0·0354  (0·0139;0·0569) |  |  |  |  |  | 0·0353  (0·0140;0·0566) |  |  |  |  | 9·5 |
| Humidity^†^ | 0·0080  (-0·0027;0·0187) | 0·3725 | 3·4 | 0·140 |  | 0·0110  (0·0005;0·0216) |  |  |  |  |  | 0·0109  (0·0005;0·0213) |  |  |  |  | 4·1 |
| Temperature^‡^ | -0·0064  (-0·0370;0·0243) | 0·3925 | -2·0 | 0·679 |  | 0·0236  (-0·0154;0·0627) |  |  |  |  |  | 0·0247  (-0·0122;0·0616) |  |  |  |  | 1·7 |
| Latitude^¥^ | 0·0084  (-0·0062;0·0231) | 0·3851 | -0·03 | 0·254 |  | 0·0236  (0·0049;0·0422) |  |  |  |  |  | 0·0232  (0·0051;0·0413) |  |  |  |  | 4·8 |

CHILDREN: Summary prevalence* (42 centres): 0·6% (95%CI: 0·5;0·7), tau2=0·3403, Q=246·98 p<0·001, I2=84·3%)

ADOLESCENTS: Summary prevalence* (60 centres): 0·8% (95%CI: 0·7;1·0), tau2=0·3850, Q=769·46 p<0·001, I2=90·5%)

HDI: Human development index (0-1). GINI: Gini index of inequality (0-100). NI: Not included in the model.

*Random effects metanalysis (restricted maximum likelihood method). ^†^Mean annual relative humidity (%). ^‡^Mean annual temperature (°C). ^¥^Absolute distance from Equator.

Web table 9. Impact of the different moderators on the variability (adjusted R^2^) of the prevalence of **eczema ever** between centres. Simple (crude) and multiple meta-regression analyses (full and best adjusted models).

|  | Crude | | | |  | Fully adjusted model | | | | |  | Best adjusted model | | | | | |
| --- | --- | --- | --- | --- | --- | --- | --- | --- | --- | --- | --- | --- | --- | --- | --- | --- | --- |
|  | Exp  (95%CI) | tau^2^ | aR^2^  (%) | p |  | aExp  (95%CI) | Model  F | Model  aR^2^  (%) | Model  tau^2^ | Model  p |  | aExp  (95%CI) | Model  F | Model  aR^2^  (%) | Model  tau^2^ | Model  p | Contribution  to aR^2^  (%) |
| **CHILDREN** | | | | | | | | | | | | | | | | | |
| HDI | 7·7924  (4·4668;11·1180) | 0·7934 | 33·80 | <0·001 |  | 6·8521  (3·0906;10·6136) | 6·25 | 39·1 | 0·73 | <0·001 |  | 6·9409  (3·7148;10·1670) | 15·5 | 41·4 | 0·7023 | <0·001 | 33·8 |
| GINI | -0·0734  (-0·1248;-0·0219) | 1·016 | 15·23 | 0·006 |  | -0·0348  (-0·0891;0·0196) |  |  |  |  |  | -0·0531  (-0·0972;-0·0089) |  |  |  |  | 7·6 |
| Humidity^†^ | 0·0096  (-0·0092;0·0284) | 1·195 | 0·26 | 0·308 |  | 0·0066  (-0·0106;0·0238) |  |  |  |  |  | N·I· |  |  |  |  |  |
| Temperature^‡^ | -0·0510  (-0·1118;0·0097) | 1·145 | 4·43 | 0·097 |  | 0·0310  (-0·0403;0·1022) |  |  |  |  |  | N·I· |  |  |  |  |  |
| Latitude^¥^ | 0·0394  (0·0117;0·0671) | 1·02 | 14·93 | 0·006 |  | 0·0213  (-0·0153;0·0578) |  |  |  |  |  | N·I· |  |  |  |  |  |
| **ADOLESCENTS** | | | | | | | | | | | | | | | | | |
| HDI | 1·4188  (-1·0936;3·9313) | 0·8165 | 0·47 | 0·263 |  | 1·7569  (-0·9445;4·4582) | 3·39 | 16·5 | 0·6848 | 0·0094 |  | 2·0245  (-0·7060;4·7751) | 3·35 | 13·5 | 0·7100 | 0·0155 | 0·5 |
| GINI | -0·0333  (-0·0614;-0·0052) | 0·7622 | 7·08 | 0·021 |  | -0·0248  (-0·0537;0·0042) |  |  |  |  |  | -0·0227  (-0·0566;0·0019) |  |  |  |  | 6·7 |
| Humidity^†^ | 0·0060  (-0·0077;0·0197) | 0·8235 | -0·38 | 0·385 |  | 0·0120  (-0·0017;0·0258) |  |  |  |  |  | N·I· |  |  |  |  |  |
| Temperature^‡^ | 0·0112  (-0·0284;0·0508) | 0·8297 | -1·13 | 0·574 |  | 0·0754  (0·0236;0·1272) |  |  |  |  |  | 0·0596  (0·0101;0·1090) |  |  |  |  | 3·0 |
| Latitude^¥^ | 0·0164  (-0·0026;0·0353) | 0·794 | 3·21 | 0·089 |  | 0·0284  (0·0032;0·0537) |  |  |  |  |  | 0·0215  (-0·0028;0·0459) |  |  |  |  | 3·3 |
| **ADULTS** | | | | | | | | | | | | | | | | | |
| HDI | 0·8242  (-2·6216;4·2700) | 0·8211 | -1·9 | 0·632 |  | 2·2438  (-0·9189;5·4064) | 4·34 | 29·0 | 0·5723 | 0·0033 |  | -0·0695  (-0·1031;-0·0359) | 9·21 | 28·4 | 0·5769 | <0·001 | 23·3 |
| GINI | -0·0615  (-0·0952;-0·0278) | 0·6182 | 23·3 | 0·001 |  | -0·0638  (-0·1034;-0·0243) |  |  |  |  |  | N·I: |  |  |  |  |  |
| Humidity^†^ | 0·0035  (-0·0135;0·0205) | 0·8226 | -2·1 | 0·683 |  | 0·0044  (-0·0120;0·0207) |  |  |  |  |  | N·I· |  |  |  |  |  |
| Temperature^‡^ | 0·0190  (-0·0276;0·0656) | 0·8122 | -0·8 | 0·414 |  | 0·0692  (0·0111;0·1273) |  |  |  |  |  | 0·0396  (-0·0009;0·0802) |  |  |  |  | 5·1 |
| Latitude^¥^ | 0·0137  (-0·0109;0·0383) | 0·8004 | 0·7 | 0·267 |  | 0·0142  (-0·0190;0·0473) |  |  |  |  |  | N·I· |  |  |  |  |  |

CHILDREN: Summary prevalence* (44 centres): 8·1% (95%CI: 6·0;10·9), tau2=1·1985, Q=13110·01 p<0·001, I2=99·61%)

ADOLESCENTS: Summary prevalence* (63 centres): 7·5% (95%CI: 6·1;9·2), tau2=0·8203, Q=9911·64 p<0·001, I2=99·4%)

ADULTS: Summary prevalence* (43 centres): 7·0% (95%CI: 5·5;9·0), tau2=0·8060, Q=8741·07 p<0·001, I2=99·7%)

HDI: Human development index (0-1). GINI: Gini index of inequality (0-100). NI: Not included in the model.

*Random effects metanalysis (restricted maximum likelihood method). ^†^Mean annual relative humidity (%). ^‡^Mean annual temperature (°C). ^¥^Absolute distance from Equator.

**Web Figure 1.** Residual plots of “current wheeze”, “symptoms of severe asthma” and “asthma ever” in children, adolescents and adults after applying the best adjusted models of the meta-regressions analyses depicted in Web tables 1-3.

**Web Figure 2**. Residual plots of “current rhinoconjunctivitis symptoms” and “symptoms of severe rhinoconjunctivitis” in children and adolescents after applying the best adjusted models of the meta-regressions analyses depicted in Web tables 4-5. (Those two markers were not available in adults and no regression which included HDI and GinI could be fitted for the common one in the three age groups i.e. “hay fever”).

**Web Figure 3**. Residual plots of “current eczema symptoms” and “eczema ever” in children and adolescents after applying the best adjusted models of the meta-regressions analyses depicted in Web tables 7 and 9. (No regression which included HDI and GinI could be fitted for “symptoms of severe eczema” in those age groups and was not available in adults. GinI was not a significant covariate in the best adjusted model for “eczema ever” in adults).

**Web Figure 4**. Regression plots of “current wheeze”, “symptoms of severe asthma” and “asthma ever” in children, adolescents and adults after applying the best adjusted models of the meta-regressions analyses depicted in Web tables 1-3.

**Web Figure 5.** Regression plots of “current rhinoconjunctivitis symptoms” and “symptoms of severe rhinoconjunctivitis” in children and adolescents after applying the best adjusted models of the meta-regressions analyses depicted in Web tables 4-5. (Those two markers were not available in adults and no regression which included HDI and GinI could be fitted for the common one in the three age groups i.e. “hay fever”).

**Web Figure 6**. Regression plots of “current eczema symptoms” and “eczema ever” in children and adolescents after applying the best adjusted models of the meta-regressions analyses depicted in Web tables 7 and 9. (No regression which included HDI and GinI could be fitted for “symptoms of severe eczema” in those age groups and was not available in adults. GinI was not a significant covariate in the best adjusted model for “eczema ever” in adults).

**Web Figure 7**. Heatmap of areas of high and low prevalence of “current wheeze”, “symptoms of severe asthma” and “asthma ever” in children, adolescents and adults after applying the best adjusted models of the meta-regressions analyses to the range of values of HDI and GinI in the study. The prevalence range of the specific disease marker is the one of the fitted values.

**Web Figure 8.** Heatmap of areas of high and low prevalence of “current rhinoconjunctivitis symptoms” and “symptoms of severe rhinoconjunctivitis” in children and adolescents after applying the best adjusted models of the meta-regressions analyses to the range of values of HDI and GinI in the study. The prevalence range of the specific disease marker is the one of the fitted values. (Both “rhinoconjunctivitis” markers were not available in adults and no regression which included HDI and GinI could be fitted for the common one in the three age groups i.e. “hay fever”).

**Web Figure 9.** Heatmap of areas of high and low prevalence of “current eczema symptoms” and “eczema ever” in children and adolescents after applying the best adjusted models of the meta-regressions analyses to the range of values of HDI and GinI in the study. The prevalence range of the specific disease marker is the one of the fitted values. (No regression which included HDI and GinI could be fitted for “symptoms of severe eczema” in those age groups and was not available in adults. GinI was not a significant covariate in the best adjusted model for “eczema ever” in adults).
